# Comprehensive Analysis of Molecular Subtypes and Hub Genes of Sepsis by Gene Expression Profiles

**DOI:** 10.3389/fgene.2022.884762

**Published:** 2022-08-12

**Authors:** Yongxing Lai, Chunjin Lin, Xing Lin, Lijuan Wu, Yinan Zhao, Tingfang Shao, Fan Lin

**Affiliations:** ^1^ Department of Geriatric Medicine, Shengli Clinical Medical College of Fujian Medical University, Fuzhou, China; ^2^ Fujian Provincial Center for Geriatrics, Fujian Provincial Hospital, Fuzhou, China

**Keywords:** sepsis, bioinformatics analysis, WGCNA, LASSO, molecular subtype

## Abstract

**Background:** Sepsis is a systemic inflammatory response syndrome (SIRS) with heterogeneity of clinical symptoms. Studies further exploring the molecular subtypes of sepsis and elucidating its probable mechanisms are urgently needed.

**Methods:** Microarray datasets of peripheral blood in sepsis were downloaded from the Gene Expression Omnibus (GEO) database, and differentially expressed genes (DEGs) were identified. Weighted gene co-expression network analysis (WGCNA) analysis was conducted to screen key module genes. Consensus clustering analysis was carried out to identify distinct sepsis molecular subtypes. Subtype-specific pathways were explored using gene set variation analysis (GSVA). Afterward, we intersected subtype-related, dramatically expressed and module-specific genes to screen consensus DEGs (co-DEGs). Enrichment analysis was carried out to identify key pathways. The least absolute shrinkage and selection operator (LASSO) regression analysis was used for screen potential diagnostic biomarkers.

**Results:** Patients with sepsis were classified into three clusters. GSVA showed these DEGs among different clusters in sepsis were assigned to metabolism, oxidative phosphorylation, autophagy regulation, and VEGF pathways, etc. In addition, we identified 40 co-DEGs and several dysregulated pathways. A diagnostic model with 25-gene signature was proven to be of high value for the diagnosis of sepsis. Genes in the diagnostic model with AUC values more than 0.95 in external datasets were screened as key genes for the diagnosis of sepsis. Finally, ANKRD22, GPR84, GYG1, BLOC1S1, CARD11, NOG, and LRG1 were recognized as critical genes associated with sepsis molecular subtypes.

**Conclusion:** There are remarkable differences in and enriched pathways among different molecular subgroups of sepsis, which may be the key factors leading to heterogeneity of clinical symptoms and prognosis in patients with sepsis. Our current study provides novel diagnostic and therapeutic biomarkers for sepsis molecular subtypes.

## Introduction

Sepsis, a systemic inflammatory response syndrome (SIRS) caused by various infectious processes, is one of the common diseases that leads to the death of hospitalized patients in the ICU (Fleischmann et al., 2016; Novosad et al., 2016). At present, we lack effective strategies for early diagnosis and treatment of sepsis due to the heterogeneity of pathogenesis and clinical symptoms in patients with sepsis (Davenport et al., 2016; Seymour et al., 2019). In addition, with the progression of sepsis and the persistence of systemic inflammation, patients with severe sepsis are commonly accompanied by multiple organ dysfunction syndromes (MODS), hypoperfusion, or hypotension, which brings a great challenge to sepsis treatment (Rello et al., 2017). Therefore, a thorough understanding of the distinct molecular subtypes of sepsis is needed to improve the prognosis of sepsis patients with different subtypes.

The release of a large number of cytokines and inflammatory mediators can result in dysregulated immune responses, which may be the decisive factor affecting the prognosis of sepsis patients (Markwart et al., 2015). In addition, the activation of abnormal genes in sepsis patients may play a critical role in the progression of the disease. In recent years, a variety of biomarkers, including serum cytokine/chemokine, cellular receptor, coagulation factors, vascular endothelial damage factors, and acute inflammatory factors have been implemented in the diagnosis and prognosis of sepsis (Pierrakos and Vincent, 2010; Sandquist and Wong, 2014). Nevertheless, because of the complexity of the pathogenesis of sepsis, the specificity and sensitivity of these biomarkers in disease diagnosis and prognosis are significantly lower than expected. Further exploring more potent biomarkers for the early diagnosis and treatment of sepsis has become urgent.

Bioinformatics analysis at the molecular biology level has been extensively applied for clinical practice by screening and predicting potential key pathways and biomarkers (Banwait and Bastola, 2015; Cheng et al., 2021). Few studies to date have explored the significance of identified molecular subtypes in the early diagnosis and treatment of diseases including cancer, respiratory diseases, and myocardial infarction (Hu et al., 2021; Li et al., 2021; Rao et al., 2021; Shi et al., 2021). Zhang et al. classified sepsis patients into three clusters based on m6A methylation regulatory genes (Zhang et al., 2020). However, whether the specific molecular subtypes could be determined based on whole genome sequencing data of sepsis patients is not yet thoroughly understood.

In this study, we classified sepsis into three molecular subtypes using unsupervised consensus clustering based on whole gene expression. Moreover, we identified consensus differentially expressed genes (co-DEGs) by intersecting the DEGs among three subtypes with differential genes screened by DEGs and WGCNA methods. Based on these results, we performed various analyses, including Gene set variation analysis (GSVA), Gene Ontology (GO), Kyoto Encyclopedia of Genes and Genomes (KEGG), Reactome enrichment analysis, Pearson correlation analysis, and protein—protein interaction (PPI) analysis. In addition, we constructed a 25-gene-based diagnosis model using least absolute shrinkage and selection operator (LASSO) regression analysis and validated their expression levels and diagnostic values for sepsis. Eventually, we identified 7 distinct hub genes among the three molecular subtypes.

## Materials

### Data Acquisition

**FIGURE 1:**
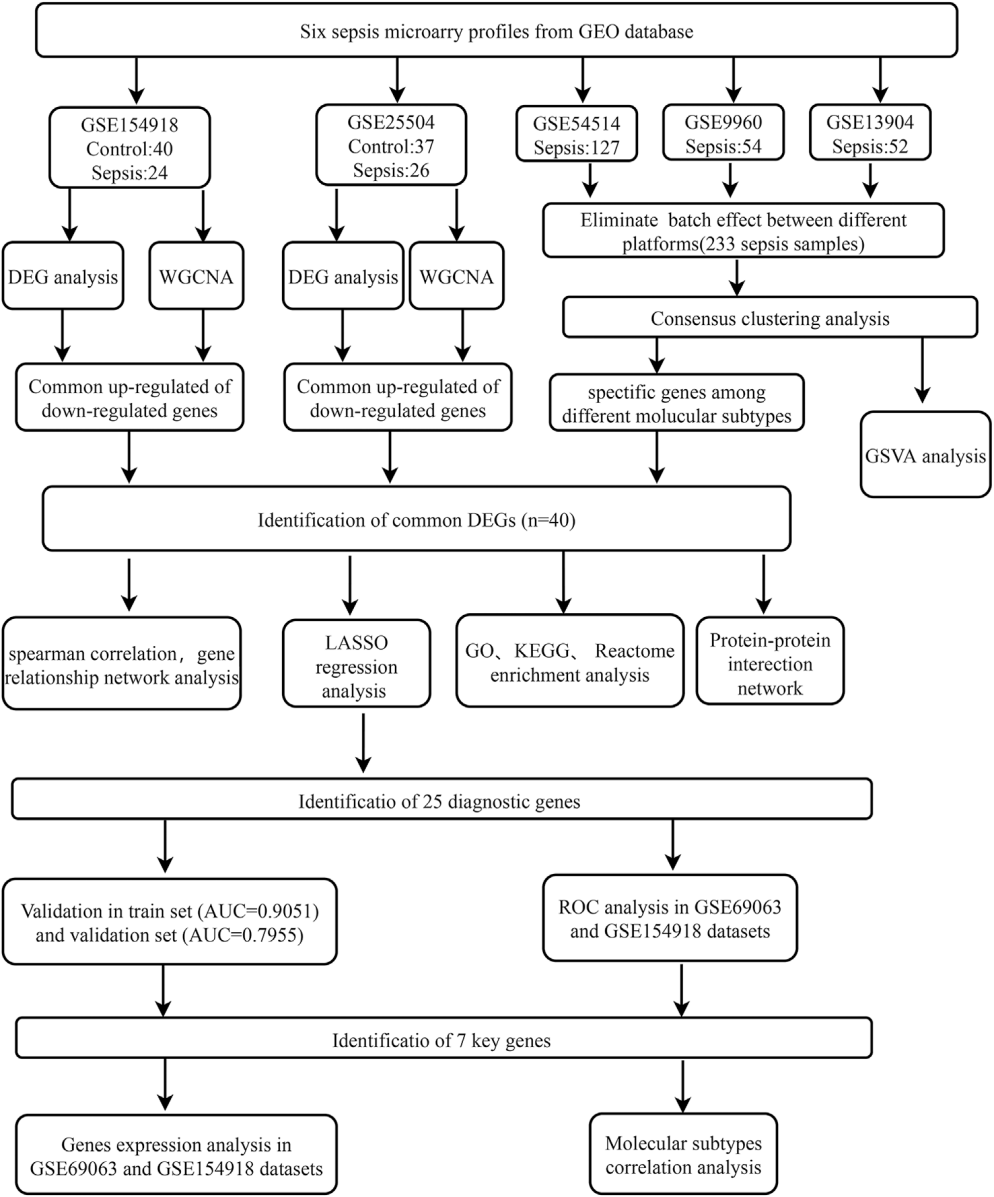
Flowchart for bioinformatics analysis in this study. The following datasets were used for screening potential diagnostic genes and mechanisms associated with the progress of sepsis (GSE154918 and GSE25504 datasets) and the sepsis molecular subtypes (GSE54514 and GSE9960, and GSE13904 datasets). Abbreviations: GEO, Gene Expression Omnibus; DEG, differentially expressed genes; WGCNA, Weighted gene co-expression network analysis; GSVA, gene set variation analysis; GO, Gene Ontology; KEGG, Kyoto Encyclopedia of Genes and Genomes; AUC, area under the curve; LASSO, The least absolute shrinkage and selection operator; ROC, Receiver operator characteristic curve.

All GEO datasets were downloaded from Gene Expression Omnibus (GEO, www.ncbi.nlm.nih.gov/geo/) (Barrett et al., 2013). We selected 6 datasets (GSE154918, GSE54514, GSE9960, GSE69063, GSE25504, and GSE13904) related to sepsis for analysis. The whole gene expression profiles of peripheral blood were extracted for further analysis. The GEO datasets collected are exhibited in Table 1. The flowchart of the study was elucidated in Figure 1.

**TABLE 1:** Information for selected microarray datasets.

GO accession	Platform	Samples	Sample source	Age	Sex (male/female)
		Control sepsis		Control sepsis	Control sepsis
GSE154918	GPL20301	40 24	Peripheral blood	-- --	17/23 10/14
GSE54514	GPL6947	36 127	Whole blood	42.94 ± 15.79 59.1 ± 16.0	12/24 52/75
GSE9960	GPL570	16 54	Peripheral blood	-- --	-- --
GSE69063	GPL20301	33 57	Peripheral blood	-- --	-- --
GSE25504	GPL570	37 26	Peripheral blood	-- --	23/14 14/12
GSE13904	GPL570	18 52	Peripheral blood	-- --	-- --

The GSE154918 dataset (GPL20301 platform) is composed of whole gene expression profiles of peripheral blood from 40 control and 24 sepsis samples, The GSE54514 dataset (GPL6947 platform) was composed of whole gene expression profiles of peripheral blood from 36 non-sepsis healthy control subjects and 127 sepsis patients, The GSE9960 dataset (GPL570 platform) was composed of whole gene expression profiles of peripheral blood from 16 control and 54 sepsis samples, The GSE69063 dataset (GPL20301 platform) was composed of whole gene expression profiles of peripheral blood from 33 control and 57 sepsis samples, The GSE25504 dataset (GPL570 platform) was composed of whole gene expression profiles of peripheral blood from 37 control and 26 sepsis samples, The GSE13904 dataset (GPL570 platform) was composed of whole gene expression profiles of peripheral blood from 18 control and 52 sepsis samples.

### DEGs Analysis

R software’s “limma” package was applied for identifying the DEGs between the sepsis and control samples in the GSE154918 and GSE25504 datasets, respectively (Ritchie et al., 2015). DEGs with |log2 fold change (FC)| > 0.5 and *p* < 0.05 were defined as statistically significant. Volcano plots and heatmaps of the identified DEGs were visualized using the “ggplot2” and “heatmap” R packages.

### Construction of the Co-Expression Network by WGCNA

R software’s “WGCNA” package was used to construct the co-expression network of the GSE154918 and GSE25504 datasets, respectively (Langfelder and Horvath, 2008). In brief, we explored the association among different pairs of genes and weighted them based on the expression levels of related genes in control and sepsis samples. Afterward, we transformed the adjacency matrix into a topological overlap matrix (TOM) to further verify the gene connectivity in the network. Next, the hierarchical clustering method was conducted to determine remarkably interconnected clusters (modules) according to gene connectivity and covariance coefficients. We selected the best power value and established the correlated modules. Finally, we performed Pearson correlation analysis and defined the highest three gene modules as statistically significant based on the correlation coefficient.

### Unsupervised Consensus Clustering Analysis

The GSE9960, GSE13904, and GSE54514 datasets were log2 transformed. The robust multi-chip average (RMA) method was utilized to normalize gene expression, and the batch effect between different platforms was eliminated using the ComBat method based on the “sva” R package. Principal component analysis (PCA) was applied for evaluating the aggregation between different samples. Afterward, R software’s “ConsensusClusterPlus” package was carried out to perform unsupervised consensus analysis (Wilkerson and Hayes, 2010) among the 233 standardized sepsis patients. The number of re-samplings settled at 1,000, with each re-sampling containing 80% of the samples. The maximum number of clusters was set to 10, and the optimal k value was determined by the cumulative distribution function (CDF) index and the consensus matrix. Finally, t-SNE was carried out to validate the subtype assignments based on the gene expression profiles of the above sepsis patients.

The dataset was normalized and summarized using robust multi-chip average (RMA) implemented in the R package affy, and batch effects were corrected.

### GSVA Analysis in Different Molecular Subtypes of Sepsis

R software’s “GSVA” and “GSEABase” packages were utilized to validate the performance of gene sets (c2. cp.kegg.v7.4. symbols) among sepsis patients with different molecular subtypes, thus identifying the enriched pathways in each subtype. Gene sets with adjusted *p*-value < 0.05 were defined as significantly enriched gene sets.

### Identification and Correlation Analysis of Co-DEGs

The DEGs were screened using DEGs analysis and the WGCNA method in GSE154918 and GSE25504 datasets, and the specific genes were also identified in sepsis patients with distinct molecular subtypes. Eventually, a total of 48 core genes were identified by intersecting all the results. The “ggVennDiagram” package was used in generating the Venn diagrams of co-DEGs.

Spearman correlation analysis was performed to determine the correlation between core genes based on the gene expression profiles. The heatmap of the correlation coefficient among these hub genes was visualized using the “corrplot” R package, and the gene relationship network diagram with a correlation coefficient >0.9 was constructed using the “igraph” R package.

### Enrichment Analysis

The Database for Annotation, Visualization, and Integrated Discovery (DAVID, https://david.ncifcrf.gov/summary.jsp), an online tool, was utilized to perform the GO enrichment analysis of co-DEGs (Huang da et al., 2009). Statistically significant GO terms (BP) with FDR <0.05 were screened and the results were visualized using the “GOplot” R package.

KEGG and Reactome enrichment analysis of these co-DEGs were conducted using the “clusterProfile” and “org.Hs.eg.db” R packages (Yu et al., 2012). The statistically significant enrichment pathways with adjusted *p*-value < 0.05 were defined and visualized using a bubble plot.

### PPI Network Analysis

The STRING database (https://strin g-db.org/) was applied for constructing the PPI network of co-DEGs, and the core genes with a combined score of more than 0.4 were screened (Szklarczyk et al., 2015). The protein–protein interaction information was visualized utilizing the Cytoscape software (version 3.8.2).

### LASSO Regression Analysis

LASSO is a regularization method with strong predictability, that is better than regression analysis when examining high-dimensional data (Bader and Hogue, 2003). A total of 303 samples including 70 health and 233 sepsis patients from GSE9960, GSE13904, and GSE54514 datasets were applied for LASSO regression analysis. As a training set, 70% of samples (N = 217, 58 controls, and 169 sepsis samples) were randomly selected. As a validation set, 30% of samples (N = 86, 22 controls, and 54 sepsis samples) were selected. The LASSO regression analysis was performed based on the expression profiles of co-DEGs using the “glmnet” R package. The predictive model constructed in the training set was verified in the testing set. In addition, receiver operating characteristic (ROC) curves were drawn in the validation set and train set using the “pROC” R package to assess the performance of the constructive model. AUC more than 0.75 was defined as a model with a high diagnostic value.

### Validation of Hub Genes

GSE154918 and GSE69063 datasets were selected as the validation set to verify the diagnostic value of these genes identified by LASSO in sepsis. Genes with AUC >0.9 in both the GSE154918 and GSE69063 were defined as the ultimate hub genes. Subsequently, we verified the expression of these hub genes in GSE154918 and GSE69063 datasets and in distinct molecular subtypes of sepsis.

## Results

### Identification of DEGs and Construction of Co-Expression Network

**FIGURE 2:**
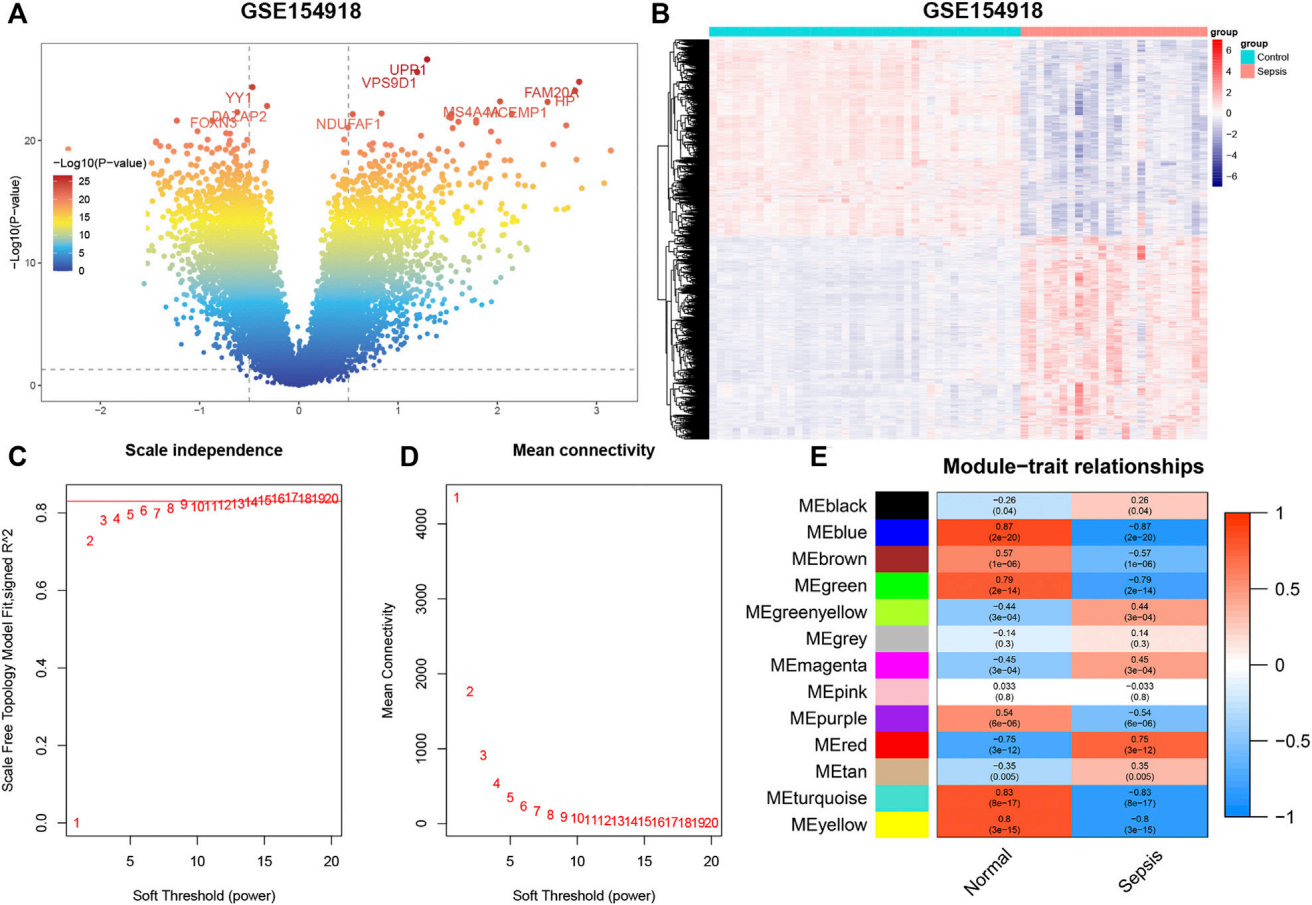
GSE154918 datasets analyzed by DEGs and WGCNA methods, respectively. **(A)** Representative volcano plot in GSE154918 dataset. **(B)** Representative heat map of DEGs between normal subjects and sepsis patients. **(C,D)** Soft threshold selection process. **(E)** Correlation of modules with clinical characteristics. Each row represents a distinct module; each column represents a distinct clinical phenotype. Red rectangle indicates positive correlation; blue rectangle indicates negative correlation.

**FIGURE 3:**
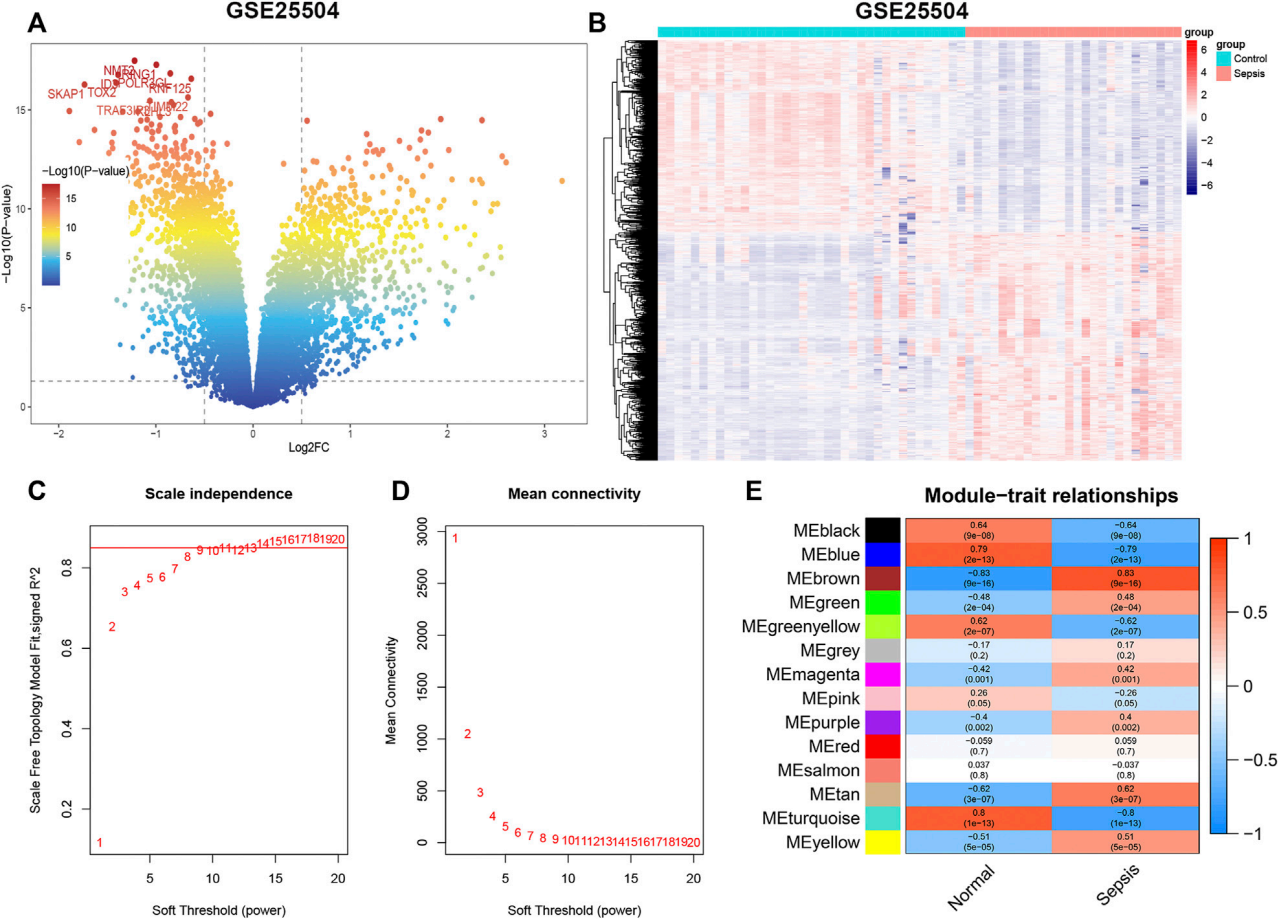
GSE25504 datasets analyzed by DEGs and WGCNA methods, respectively. **(A)** Representative volcano plot in GSE25504 dataset. **(B)** Representative heat map of DEGs between normal subjects and sepsis patients. **(C,D)** Soft threshold selection process. **(E)** Correlation of modules with clinical characteristics. Each row represents a distinct module; each column represents a distinct clinical phenotype. Red rectangle indicates positive correlation; blue rectangle indicates negative correlation.

The flowchart of our study is shown in Figure 1. To identify sepsis-related genes, we firstly analyzed the DEGs between sepsis and control samples in the GSE154918 dataset. A total of 1,671 up-regulated and 1,623 down-regulated genes were determined using the DEGs method (Figures 2A,B, Supplementary Figure S1A). Subsequently, we applied the WGCNA method to study the co-expression network in the GSE154918 dataset. When the best soft threshold power settled at 6, the mean connectivity was more effective (Figures 2C,D). A total of 13 co-expressed gene modules were created. among which the blue (4,036 genes, R = 0.87, *p* < 0.05), turquoise (4,618 genes, R = 0.83, *p* < 0.05), and yellow modules (1719 genes, R = 0.8, *p* < 0.05) had the most correlation with sepsis patients (Figure 2E, Supplementary Figure S1B **)**. In addition, the blue (R = 0.85, *p* < 0.05), turquoise (R = 0.95, *p* < 0.05), and yellow (R = 0.92, *p* < 0.05) modules were significantly correlated with module-related genes (Supplementary Figures S1C–H). Meanwhile, we identified 945 up-regulated and 793 down-regulated genes using the DEGs method in the GSE25504 dataset (Figures 3A,B, Supplementary Figure S2A). Based on the WGCNA method, a relatively high mean connectively was maintained by setting the best soft threshold power to 8 (Figures 3C,D). We clustered a total of 14 co-expressed gene modules, among which the blue (2,174 genes, R = 0.79, *p* < 0.05), brown (1717 genes, R = -0.83, *p* < 0.05), and turquoise (2,478 genes, R = 0.8, *p* < 0.05) modules were closely correlated with sepsis patients (Figure 3E, Supplementary Figure S2B)**.** Moreover, blue (R = 0.88 *p* < 0.05), brown (R = 0.93, *p* < 0.05), and turquoise (R = 0.9, *p* < 0.05) modules were also remarkably correlated with module-related genes (Supplementary Figures S2C–H).

### Consensus Clustering Analysis for Sepsis

**FIGURE 4:**
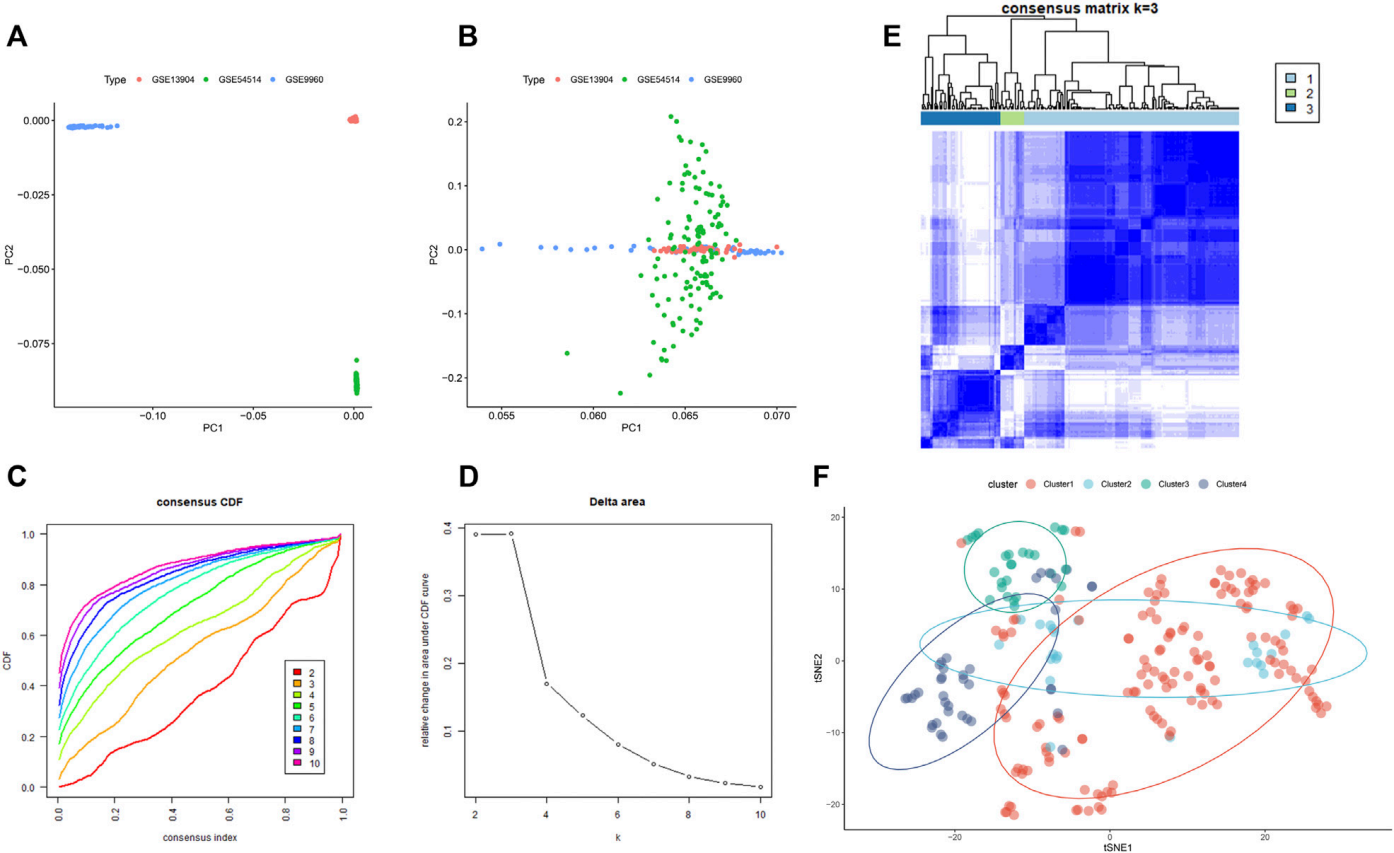
The consensus clustering analysis of sepsis patients. **(A)** Representative PCA clustering diagram of GSE13904, GSE54514, and GSE9960 datasets before batch correction. **(B)** Representative PCA clustering diagram of GSE13904, GSE54514, and GSE9960 datasets after batch correction. **(C)** Cumulative distribution function (CDF) curves of clustering (k, 2–10). **(D)** The CDF Delta area curve of all sepsis samples when k = 2–10. **(E)** Consistency matrix of the sample with k = 3. **(F)** t-SNE confirmed the classification of three clusters: Cluster1, Cluster3, and Cluster4.

The GSE9960, GSE13904, and GSE54514 datasets were mixed into a combined dataset containing a total of 233 sepsis samples. These three datasets exhibited obvious separation before batch correction (Figure 4A). While the batch effect among these datasets from different platforms had been successfully eradicated after batch correction (Figure 4B). Then, we performed molecular subtypes analysis in a combined dataset based on the expression of genes via the “ConsensusClusterPlus” R package. Clustering results suggested that the classification was most reliable and stable when k = 3 (Figures 4C–E, Supplementary Figure S3). Consistently, the t-SNE confirmed that only cluster1, cluster3, and cluster4 could be significantly separated (Figure 4F). In total, 233 sepsis patients were classified into three subtypes, including cluster1 (n = 144), cluster 3 (n = 26), and cluster4 (n = 39) based on gene expression levels, which were chosen for subsequent analysis.

### Identification of the DEGs Between Different Clusters in Sepsis

The DEGs among different molecular subtypes of sepsis patients were identified based on whole genome expression profiles. Eight hundred and sixty-seven (473 up-regulated and 394 down-regulated) DEGs were identified between cluster 1 and cluster 3 samples (Supplementary Figures S4A,B)**.** The top 10 overexpressed and suppressed DEGs between cluster 1 and cluster 3 were exhibited in Table 2 **.** In addition, a total of 420 (237 up-regulated and 183 down-regulated) DEGs were screened after comparing cluster 1 with cluster 4 samples (Supplementary Figures S4C,D). The top 10 overexpressed and suppressed DEGs between cluster 1 and cluster 4 were exhibited in Table 3. Moreover, one thousand four hundred and forty-five (696 up-regulated and 749 down-regulated) DEGs were acquired after comparing cluster 3 with cluster 4 samples (Supplementary Figures S4E,F). The top 10 overexpressed and suppressed DEGs between cluster 3 and cluster 4 were exhibited in Table 4 **.** Overall, we identified 1803 specific genes among three subgroups.

**TABLE 2:** The top 10 upregulated and downregulated DEGs between cluster 1 and cluster 3.

Gene	logFC	AveExpr	*P*.Value	adj.P.Val
LOR	2.232911	7.365389	2.44E-17	6.25E-15
MATN3	2.116437	7.342705	1.27E-33	1.73E-29
ACER1	1.917453	7.134010	1.00E-10	2.89E-09
EIF3M	1.915094	9.821831	3.91E-21	4.70E-18
EGR1	1.885098	7.298329	7.11E-14	5.97E-12
FCGR2B	1.867446	9.233309	1.33E-10	3.76E-09
ADGRL2	1.825619	7.841062	4.86E-20	2.64E-17
MMS19	1.810922	8.032800	2.60E-17	6.56E-15
HSP90B1	1.781601	7.969837	8.66E-20	4.53E-17
CYB561D1	1.723145	8.652701	1.58E-15	2.26E-13
PDE7B	−2.184322	6.682677	1.19E-15	1.82E-13
ICOSLG	−2.128255	6.694072	6.11E-08	8.27E-07
COX8C	−1.786688	6.581754	7.79E-12	3.06E-10
LRG1	−1.708443	7.463786	3.90E-19	1.66E-16
BATF2	−1.655835	6.366884	1.05E-08	1.75E-07
CDSN	−1.411182	7.786637	2.14E-05	0.000139
N4BP2L2-IT2	−1.329963	6.741250	6.64E-12	2.71E-10
EGLN1	−1.318017	8.282652	1.35E-13	1.04E-11
GSX1	−1.303214	7.582201	4.58E-21	4.80E-18
	−1.205521	6.025581	1.41E-14	1.47E-12

**TABLE 3:** The top 10 upregulated and downregulated DEGs between cluster 1 and cluster 4.

Gene	logFC	AveExpr	*P*.Value	adj.P.Val
BDNF	1.313856221	7.02288789	8.53E-11	3.09E-09
LOC414300	1.259871113	6.136214095	2.16E-27	2.94E-23
MMS19	1.201391888	8.032799817	8.06E-12	4.44E-10
LOC389895	1.192141492	6.291234077	9.92E-18	8.43E-15
HEPACAM2	1.142216053	8.393840633	1.20E-11	6.18E-10
CELF3	1.127665021	7.986695369	6.40E-12	3.71E-10
PCDHGA1	1.109262127	6.187973661	1.40E-12	1.03E-10
MGAT4EP	1.100499218	7.157119305	9.50E-11	3.37E-09
LOR	1.095497243	7.365389255	2.13E-07	2.37E-06
LRFN1	1.085804828	6.963573766	7.25E-14	1.02E-11
CDC23	−1.19332674	6.623615158	1.49E-10	4.86E-09
COQ10A	−1.169422871	7.020004172	5.69E-09	1.06E-07
CFAP44	−1.16466466	6.534720358	1.05E-09	2.49E-08
GPC6	−1.161193892	6.730830401	4.04E-10	1.11E-08
DSCAML1	−1.121255594	5.706122812	6.82E-11	2.56E-09
LINC01127	−1.060769752	6.398435749	1.19E-16	5.61E-14
AMPD2	−1.04287083	6.47352482	2.51E-09	5.23E-08
DDAH2	−0.964605107	7.06749405	1.09E-12	8.73E-11
ARL13B	−0.955187663	6.191602504	6.72E-11	2.54E-09
HTR2A	−0.947381679	5.887182929	2.34E-11	1.07E-09

**TABLE 4:** The top 10 upregulated and downregulated DEGs between cluster 3 and cluster 4.

Gene	logFC	AveExpr	*P*.Value	adj.P.Val
PDE7B	2.37419879	6.682677353	1.28E-13	5.62E-12
N4BP2L2-IT2	2.225680688	6.741249953	3.00E-20	1.46E-17
COX8C	2.026111637	6.581753606	5.35E-11	1.09E-09
BATF2	1.728054759	6.366884467	3.99E-07	3.05E-06
HPGD	1.702575221	6.547997156	1.43E-11	3.42E-10
BOD1L1	1.687934805	8.282667200	1.98E-16	1.93E-14
LRG1	1.66910917	7.463785788	3.90E-14	1.97E-12
ICOSLG	1.633042894	6.694071983	0.000358736	0.001382
CCL22	1.625598883	6.434988283	1.54E-07	1.31E-06
GPI	1.60184548	8.380121891	2.24E-20	1.17E-17
ACER1	−2.257612424	7.134010446	1.42E-10	2.61E-09
C12orf66	−1.93072651	7.424147774	3.03E-22	5.90E-19
DSCAML1	−1.889688676	5.706122812	1.58E-14	8.82E-13
FAHD2CP	−1.87195289	6.777812514	7.42E-08	6.85E-07
CFAP44	−1.648897199	6.534720358	7.51E-10	1.12E-08
GPC6	−1.576279685	6.730830401	1.29E-09	1.81E-08
COQ10A	−1.540559836	7.020004172	3.87E-08	3.79E-07
LINC01127	−1.517861613	6.398435749	3.59E-17	4.44E-15
C1QC	−1.51682708	6.781529523	7.06E-10	1.06E-08
PCDHA2	−1.48841099	5.776736054	8.34E-13	2.80E-11

### GSVA Analysis in Different Molecular Subtypes of Sepsis

**FIGURE 5:**
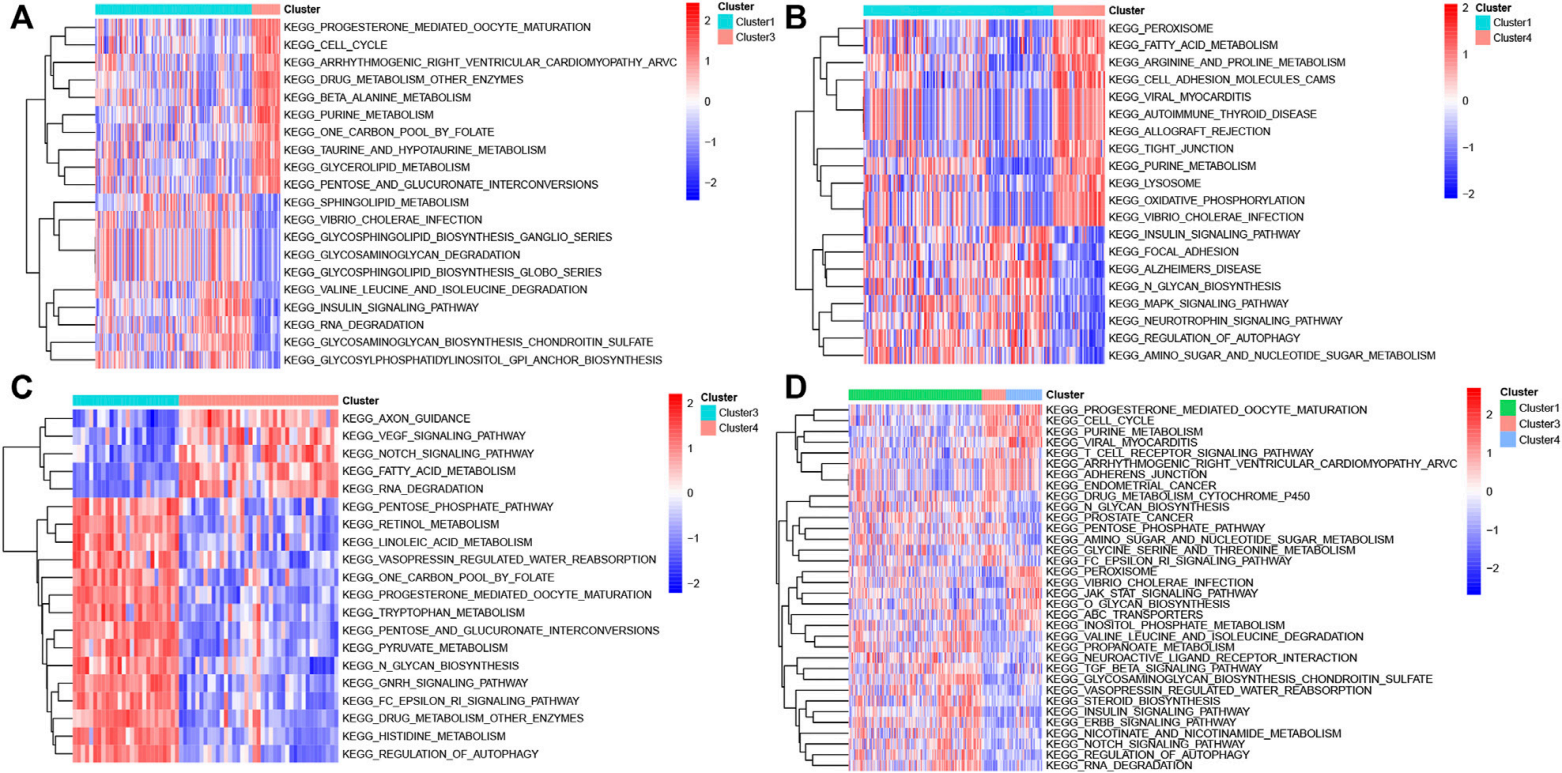
GSVA analysis in different molecular subtypes of sepsis. **(A)** Representative heat map of the top 20 differentially enriched gene pathways between Cluster1 and Cluster3. **(B)** Representative heat map of the top 20 differentially enriched gene pathways between Cluster1 and Cluster4. **(C)** Representative heat map of the top 20 differentially enriched gene pathways between Cluster3 and Cluster4. **(D)** Representative heat map of the differentially enriched gene pathways among distinct clusters.

GSVA analysis was performed among distinct molecular subtypes of sepsis patients based on the expression profiles of DEGs. In total, 103 differentially enriched gene pathways, comprising 59 pathways with activation and 44 pathways with suppression were screened when comparing cluster1 with cluster3 samples (Supplementary Table S1). The top 20 differentially enriched gene pathways between cluster1 and cluster3 are exhibited in a heatmap (Figure 5A). A total of 90 differentially enriched gene pathways, consisting of 42 pathways with activation and 48 pathways with suppression, were obtained when comparing cluster1 with cluster4 samples (Supplementary Table S2)**.** The top 20 differentially enriched gene pathways between cluster1 and cluster4 are exhibited in a heatmap (Figure 5B). A total of 110 differentially enriched gene pathways were obtained, including 56 pathways with activation and 54 pathways with suppression after comparing cluster3 with cluster4 samples (Supplementary Table S3)**.** The top 20 differentially enriched gene pathways between cluster3 and cluster4 are exhibited in a heatmap (Figure 5C). Finally, a total of 34 enriched gene pathways among clusters were identified and presented in a heatmap (Figure 5D).

### Identification and Enrichment Analysis of Co-DEGs

**FIGURE 6:**
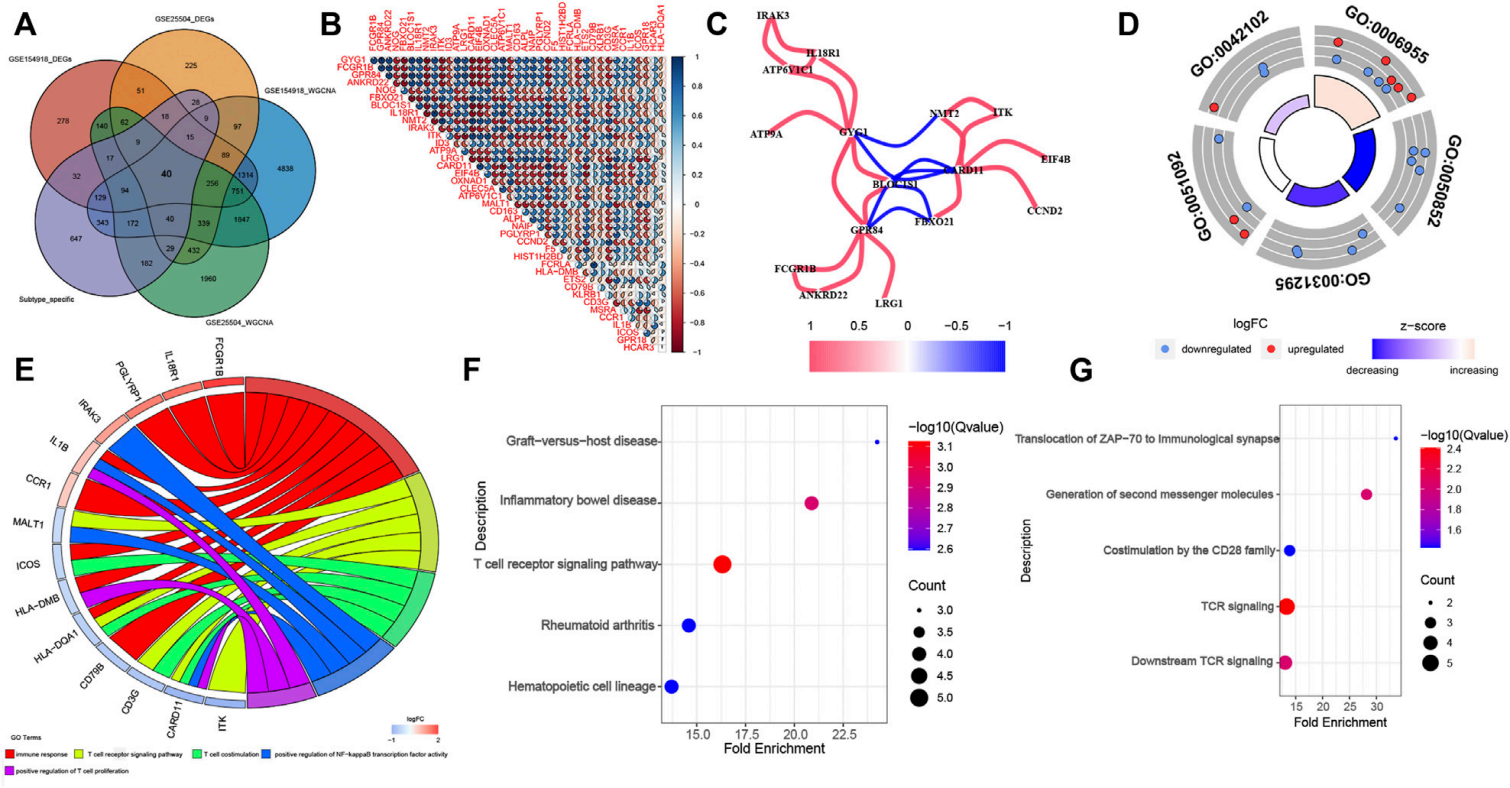
Identification of co-DEGs and screening enriched pathways related to co-DEGs. **(A)** Representative Venn diagram of intersection genes. **(B)** Representative correlation coefficient heat map of 40 co-DEGs. **(C)** Representative diagram of gene relationship network with a correlation coefficient of more than 0.9. **(D)** The first 5 GO enrichment analysis (BP) results of the 40 co-DEGs. **(E)** Representative chord plot of the first 5 GO enrichment analysis (BP) results of the 40 co-DEGs. **(F,G)** The top 5 KEGG **(F)** and Reactome **(G)** enrichment analysis results of the 40 co-DEGs.

The DEGs identified by the DEGs and WGCNA methods in GSE154918 and GSE54514 datasets were intersected with the specific genes among different molecular subtypes of sepsis patients. Eventually, a total of 40 co-DEGs were screened (Figure 6A). Spearman’s correlation analysis was performed to illuminate the correlation patterns among these 40 co-DEGs, and the area of the pie chart represents the exact value of correlation coefficients. We found a significant correlation among these co-DEGs. For example, GPR84 show strong antagonistic effects with GYG1 (coefficient = 0.968), FCGR1B (coefficient = 0.968), and ANKRD22 (coefficient = 0.912). Simultaneously, CARD11 could also present synergistic effects with GYG1 (coefficient = -0.908), GPR84 (coefficient = -0.91), and BLOC1S1 (coefficient = -0.918) (Figure 6B). These results suggested the regulatory balance among these co-DEGs. In addition, the gene relationship network indicated that genes with a correlation coefficient of more than 0.9 were as follows: IRAK3, IL18R1, ATP6V1CA, GYG1, ATP9A, NMT2, ITK, EIF4B, CCND2, CARD11, FBXO21, BLOC1S1, GPR84, LRG1, ANKRD22, FCGR1B (Figure 6C), indicating that the molecular subtypes of sepsis may be the results of multi-gene interactions**.** Subsequently, we assessed the enrichment pathways in which these co-DEGs involve. The GO enrichment analysis suggested that these co-DEGs were primarily enriched in immune-related biological functions and pathways, such as immune response, T cell receptor signaling pathway, T cell costimulation, positive regulation of NF-kappaB transcription factor activity 4, positive regulation of T cell proliferation (Table 5 and Figures 6D,E). KEGG pathway enrichment analysis indicated that co-DEGs chiefly participated in autoimmune and inflammation-related diseases including Graft−versus−host disease, Inflammatory bowel disease, and Rheumatoid arthritis. Additionally, Immune responses and hematopoietic-related signaling pathways such as T cell receptor signaling pathway and Hematopoietic cell lineage were also closely associated with these co-DEGs (Figure 6F). Consistently, Reactome enrichment analysis also revealed that these co-DEGs were mainly implicated in immunomodulation and signal transduction such as Translocation of ZAP-70 to immunological synapse, Generation of second messenger molecules, Costimulation by the CD28 family, TCR signaling, and Downstream TCR signaling (Figure 6G). Combining these results, it can be inferred that abnormal immune function may be the critical pathogenesis of sepsis. In addition, the PPI network of 40 co-DEGs, including 22 nodes and 27 edges, was analyzed by the STRING website and visualized by the Cytoscape software (Supplementary Figure S5).

**TABLE 5:** Top 5 GO terms (BP) of the 40 co-DEGs with the DAVID analysis.

ID	Term	Count	Genes	Fold	FDR
				Enrichment	
GO:0006955	immune response	9	CCR1, CD79B, HLA-DMB, IL1B, PGLYRP1, ICOS, FCGR1B, IL18R1, HLA-DQA1	9.2045	3.69E-06
GO:0050852	T cell receptor signaling pathway	5	ITK, CD3G, CARD11,MALT1, HLA-DQA1	14.5461	3.39E-04
GO:0031295	T cell costimulation	4	CD3G, ICOS, CARD11, HLA-DQA1	22.0802	7.24E-04
GO:0051092	positive regulation of NF-kappaB transcription factor activity	4	IL1B, IRAK3, CARD11, MALT1	12.9493	3.35E-03
GO:0042102	positive regulation of T cell proliferation	3	HLA-DMB, IL1B, CARD11	21,5282	8.13E-03

### Identification of Potential Biomarkers Associated With Sepsis Using LASSO Regression

**FIGURE 7:**
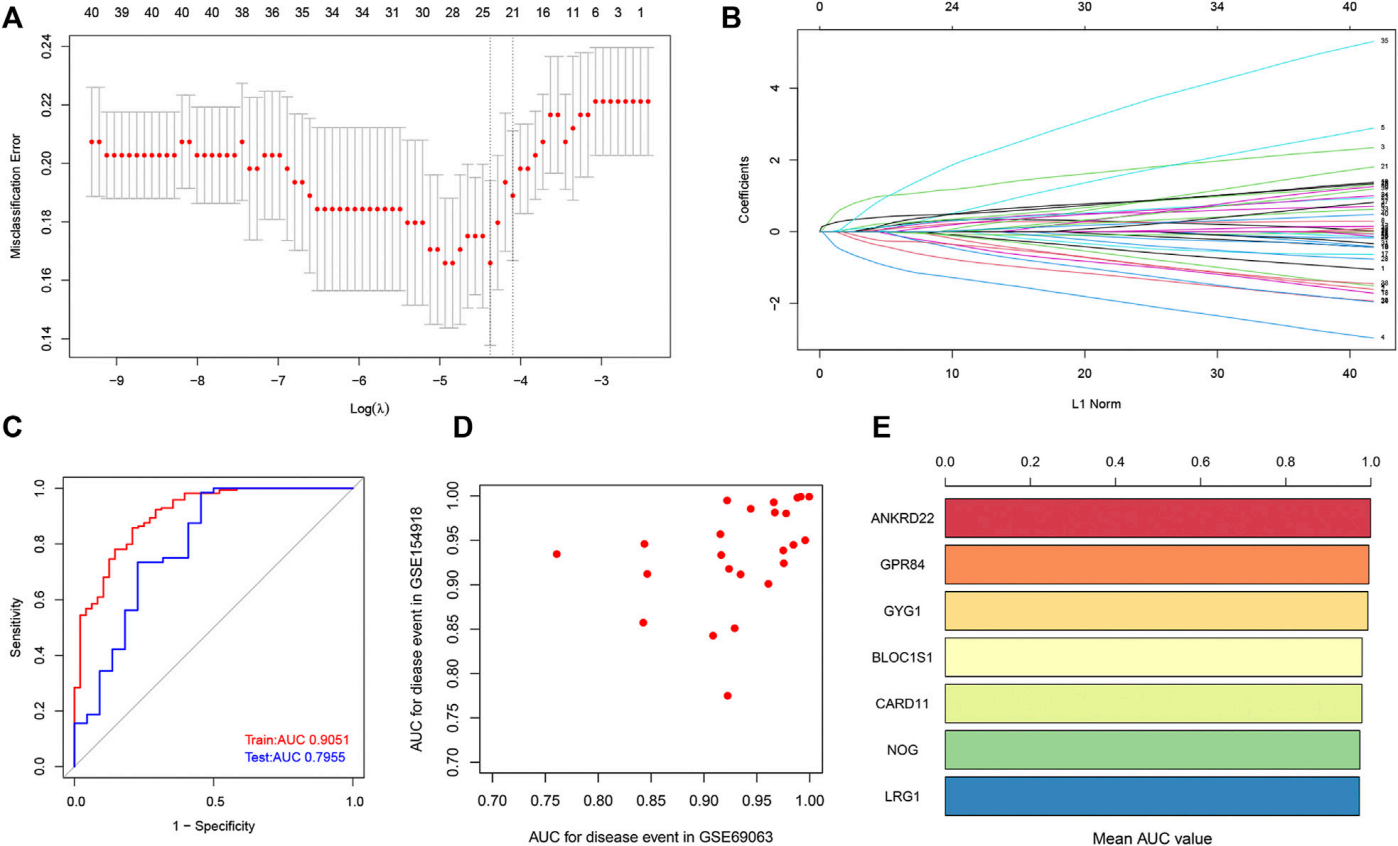
Identification of potential core genes through the LASSO model. **(A)** Selection of potential gene signature based on the optimal parameter (lambda) in the LASSO regression model. **(B)** The LASSO coefficient profiles of DEGs identified by the optimal lambda. **(C)** ROC curve analysis in train dataset and test dataset. **(D)** Potential genes with the value of AUC in both GSE154918 and GSE69063 datasets are shown in a scatter plot. **(E)** Genes with a value of AUC more than 0.95 in both GSE154918 and GSE69063 datasets are shown in a bar chart.

To further screen out potential biomarkers for sepsis, we performed the LASSO regression analysis based on the expression profile of co-DEGs. We separated a total of 303 samples in the combined dataset (GSE9960, GSE13904, and GSE54514 datasets, 233 sepsis and 70 control samples) into a training set (70%) and a validation set (30%). Eventually, a total of 25 potential biomarkers with a non-zero coefficient were obtained (Figures 7A,B). The AUC of the 25-gene signature was 0.9051 in the training set and 0.7955 in the validation set (Figure 7C), which suggests that the 25-gene-based model may be able to correctly diagnose sepsis. Subsequently, we also utilized external datasets to ascertain the diagnostic value of these potential biomarkers. The value of the AUC of these potential biomarkers in the GSE154918 and GSE69063 datasets were exhibited in a scatter plot (Figure 7D). Genes with AUC >0.95 in both the GSE154918 and GSE69063 datasets were as follows: ANKRD22, GPR84, GYG1, BLOC1S1, CARD11, NOG, and LRG1 (Figure 7E). These results reveal that the 7 core genes identified have the most ability to differentiate sepsis from healthy controls.

### Verification of the Hub Genes Expression

**FIGURE 8:**
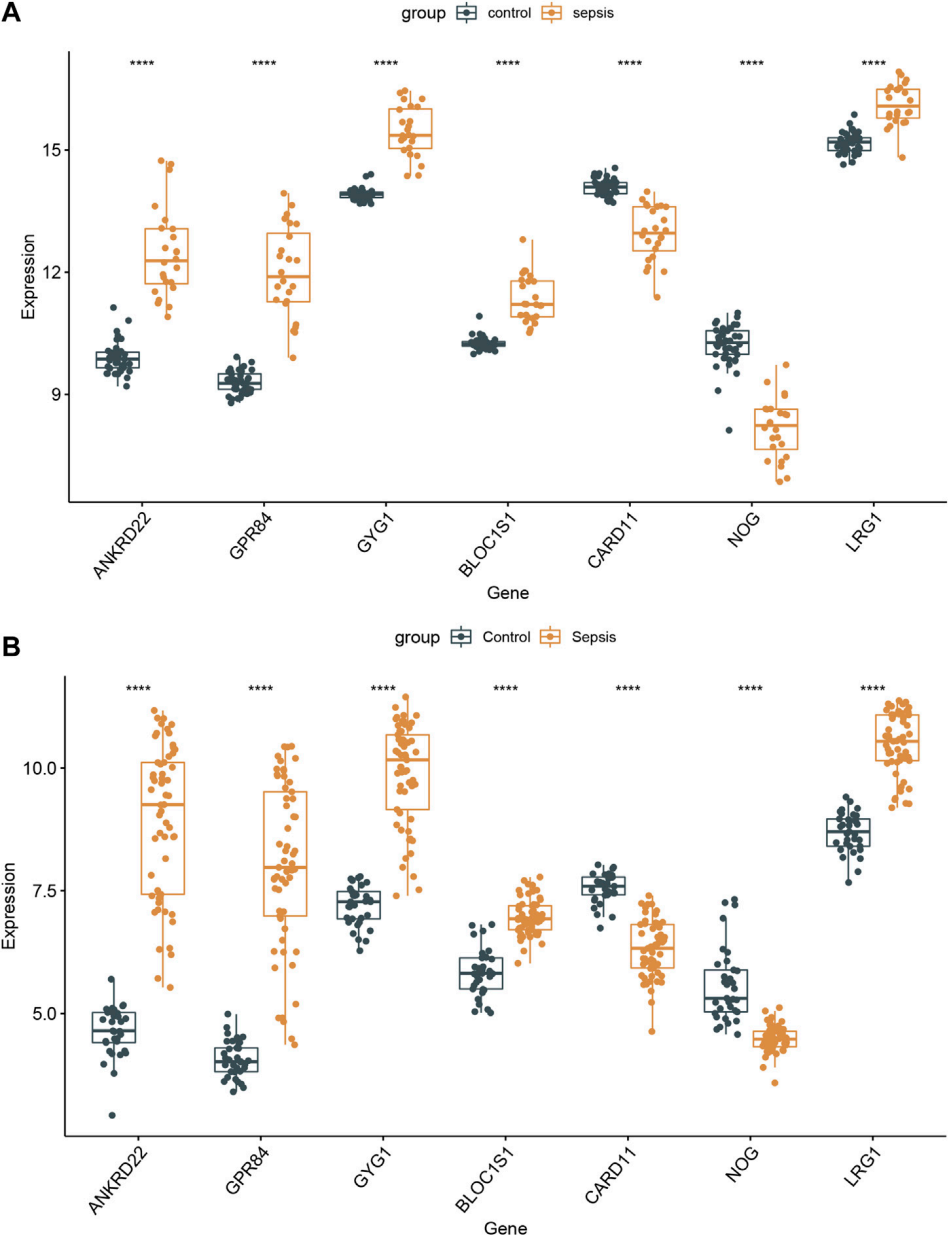
Validation of the 7 specifically expressed hub genes. **(A)** Verification of the expression of 7 specifically hub genes in the GSE154918 dataset. **(B)** Verification of the expression of 7 specifically hub genes in different sepsis molecular subtypes.

To further demonstrate whether these 7 hub genes were worth using in clinical practice, we validated the expression of these core genes in different datasets. In GSE154918 and GSE69063 datasets, ANKRD22, GPR84, GYG1, BLOC1S1, and LRG1 were all highly expressed in sepsis patients, whereas NOG and CARD11 were dramatically decreased in sepsis patients (Figures 8A,B). Meanwhile, these 7 core genes were also subtype-specific biomarkers. Therefore, we speculated that these 7 hub genes have a high diagnostic ability in sepsis patients and are closely related to different subtypes of sepsis.

## Discussion

Sepsis is a life-threatening inflammatory response syndrome caused by an unbalanced response of the host to various infection processes (van der Poll et al., 2017). Due to a lack of timely early diagnosis and treatment, sepsis has become one of the diseases with a high fatality and disability rate worldwide, and reducing the mortality rate has become the ultimate goal of its treatment. With in-depth studies of the pathogenesis of sepsis and the continuous attempt at treatment methods, distinct biomarkers have been employed in the diagnosis and treatment monitoring of sepsis (Faix, 2013; Nguyen et al., 2016). However, the interaction of the multiple genes involved in diverse biological functions may cause individual differences and complex pathophysiological mechanisms of sepsis (Maslove and Wong, 2014), which leads to unsatisfactory clinical diagnosis of a single biomarker. Therefore, it is urgent to identify distinct molecular subtypes of sepsis and elucidate the core genes and pathways associated with sepsis.

In our current study, we identified the DEGs and specific gene modules associated with sepsis in GSE154918 and GSE25504 datasets, utilizing the DEGs and WGCNA methods, respectively. Moreover, the “Consensus ClusterPlus” package was used to unsupervised cluster the patients of 233 sepsis patients from the GSE9960, GSE13904, and GSE54514 datasets. Afterward, a total of 40 co-DEGs were obtained by intersecting the DEGs, specific gene modules, and molecular cluster-related genes. GO, KEGG and Reactome enrichment analysis of these co-DEGs all indicated that sepsis was closely related to immune response and signal transduction, which may be the primary factor leading to sepsis progression. Therefore, screening of co-DEGs and identification of enrichment pathways significantly reduced the scope of our research, which may be able to ascertain more effective biomarkers for the early diagnosis and treatment of sepsis.

Defining different cut-off values plays a decisive role in DEG analysis. It is widely recognized that a more stringent screening criterion makes the results more convincing. However, according to the strict criterion for DEGs analysis (adjusted P-value < 0.05 and |logFC| > 1) (Liu et al., 2021; Lu et al., 2021; Su et al., 2021), we only obtained a total of 208 up-regulated and 380 down-regulated genes in the GSE154918 datasets, and 119 up-regulated and 302 down-regulated genes in the GSE25504 dataset. Therefore, we selected relatively rough cut-off values (*p*-value < 0.05 and |logFC| > 0.5) to identify as many DEGs and co-DEGs as possible. We finally acquired 3,294 DEGs, including 1,671 up-regulated and 1,623 down-regulated genes in the GSE154918 dataset, and 1738 DEGs, including 945 up-regulated and 793 down-regulated genes in the GSE25504 dataset.

Different sepsis patients have distinct prognoses, which may be caused by multiple pathways in which different key genes are involved. In our study, we performed an unsupervised cluster for sepsis patients based on whole-genome expression profiles and clustered 233 sepsis patients into three molecular subtypes. However, only 79 overlapped cluster-specific DEGs were screened when overlapping these DEGs among three subgroups. Therefore, to identify as many cluster-specific DEGs as possible, we merged these results and applied these 1803 merged cluster-specific DEGs for subsequent analysis. The consequences of GSVA analysis indicated that 103 differentially enriched gene pathways between cluster1 and 3 were screened, 90 differentially enriched gene pathways between clusters 1 and 4 were identified, and 110 differentially enriched gene pathways between clusters 1 and 4 were screened. These enriched gene pathways, such as metabolism, oxidative phosphorylation, autophagy regulation, and VEGF pathways have proved to be associated with the sepsis prognosis. Metabolism, the chemical reaction necessary for cell survival, usually retains the homeostasis between anabolism and catabolism under normal conditions (Qiu P. et al., 2019; Judge and Dodd, 2020; Xu et al., 2021). Additionally, metabolism involves the regulation of diverse cellular pathways, thus offering a large amount of energy for cells to ensure the execution of the function (Mulukutla et al., 2016; Panda, 2016). Oxidative phosphorylation is a coupling reaction that usually occurs in the mitochondrial inner membrane, which is characterized by the generation of cellular ATP based on mitochondrial electron transfer reactions (Ashton et al., 2018; Nolfi-Donegan et al., 2020; Głombik et al., 2021). Targeting mitochondrial oxidative phosphorylation is generally considered to be a novel strategy for the treatment of sepsis. Autophagy has been widely explored in numerous diseases including sepsis (Levy, 2007; Su et al., 2019). As previously described, autophagy exerts effects on immune regulation and inhibition of tissue damage after sepsis through regulating the expression of various immune cells (Oami et al., 2017). Moreover, autophagy has been demonstrated as an effective target for alleviating oxidative stress-induced organ failure after sepsis (Thiessen et al., 2017). VEGF pathways mainly participated in the regulation of blood vessel growth and play a vital role in promoting endothelial proliferation, migration, and survival by preserving the homeostasis of microvasculature (Ferrara and Gerber, 2001; Breen, 2007; Apte et al., 2019). With the progression of sepsis, VEGF activation results in vascular leak and dysfunction of host response, eventually leading to sepsis-related hypotension (van der Flier et al., 2005; Schuetz et al., 2011). To further screen sepsis-related hub genes, we intersected the DEGs identified by the DEGs and WGCNA methods with subtypes-related specific genes. A total of 40 co-DEGs were identified, most of which existed with significant correlation. The results of GO, KEGG, and Reactome enrichment analysis revealed that the immune response may be closely connected with the severity of sepsis. Therefore, we speculated that the interaction of immune response with autophagy, VEGF, oxidative stress, and metabolic pathways may be the major factor leading to the progression of sepsis (Minion and Tewari, 2018; Yin et al., 2019; McBride et al., 2020; Huff et al., 2021). These results, combined with our findings, indicate that these differentially activated pathways could develop potential therapeutic targets for sepsis patients with distinctive molecular subtypes.

In our current study, we received a 25-gene signature based on the LASSO model, which can accurately diagnose sepsis in both the train and validation datasets. Among them, ANKRD22, GPR84, GYG1, BLOC1S1, CARD11, NOG, and LRG1 were further identified as the most relevant key genes based on AUCs in the GSE154918 and GSE69063 datasets. Bioinformatics analysis demonstrated that ANKRD22 and NOG can serve as potential biomarkers for the progression of cancers (Tarragona et al., 2012; Qiu Y. et al., 2019; Wu et al., 2021). GPR84 is a kind of G-protein-coupled receptor activated by free fatty acids (FFA) and plays a critical role in regulating lipid metabolism (Paulsen et al., 2014; Recio et al., 2018). Enhanced GPR84 is closely related to the activation of inflammation, thus exacerbating the development of adiposity and diabesity (Nagasaki et al., 2012). GYG1 deficiency is known to be associated with polyglucosan body myopathy (Malfatti et al., 2014) and BLOC1S1 is widely recognized as a degradation substrate for IRE1alpha (Hur et al., 2012). Mutations in CARD1, a protein carrying special caspase-related recruitment domains, can result in the poor prognosis of diffuse large B-cell lymphoma (Dong et al., 2020). LRG1, which serves as a novel angiogenic factor, is required for the regulation of pathogenic angiogenesis (Wang et al., 2017). Although no previous studies have reported on the association between these 7 key genes and sepsis, the results of our analysis indicate that these hub genes may be potential markers for early diagnosis of sepsis.

To further explore and verify the clinical application value, we validated the expression levels of these 7 core genes in external datasets. All of the genes varied significantly between normal and sepsis samples in the GSE154918 and GSE69063 datasets. Additionally, sepsis with different molecular subtypes also exhibited the distinct expression of these 7 core genes. Further research needs to be carried out to elucidate the molecular mechanism of the 7 hub genes involved in the pathogenesis of sepsis molecular subtypes.

Several limitations have to be taken into account in our current study. First, the datasets used for analyses contained different sample sizes of normal and sepsis patients, which may influence the accuracy of the analytical results. Second, these datasets were downloaded from a publically available database, and lacked information on main clinical features such as sex, age, complications, recurrence rate, and individual therapeutic effect. A supplementary study is required, with a more detailed analysis of the demographic and clinical characteristics of sepsis. In addition, relatively rough cut-off values (*p*-value < 0.05 and |logFC| > 0.5) may influence the accuracy of the results. Moreover, the results of our analysis need to be confirmed *in vitro*, *in vivo*, and in clinical trials studies.

## Conclusion

In conclusion, we identified 40 co-DEGs and several immune response pathways related to sepsis prognosis using various bioinformatics analyses. We constructed a 25-gene signature diagnostic model based on LASSO regression analysis, which has a high value for the early diagnosis of sepsis. There are remarkable differences in ANKRD22, GPR84, GYG1, BLOC1S1, CARD11, NOG, and LRG1 gene expression and enriched pathways among different molecular subgroups of sepsis, which may be the key factors leading to the heterogeneity of clinical symptoms and prognosis in patients with sepsis. Our current study provides novel diagnostic and therapeutic biomarkers for sepsis molecular subtypes.

## Data Availability

The dataset(s) supporting the conclusions of this article are available in the [GSE datasets] repository, [https://www.ncbi.nlm.nih.gov/geo/], with the following data accession identifier(s): GSE154918, GSE54514, GSE9960, GSE69063, GSE25504, GSE13904.
