# Supplementary material for: Comprehensive Analysis of Molecular Subtypes and Hub Genes of Sepsis by Gene Expression Profiles
Source: Front Genet. 2022 Aug 12;13:884762. doi: 10.3389/fgene.2022.884762 (PMC9412106; doi:10.3389/fgene.2022.884762)
Supplement: Supplementary file 1 [file Table1.DOCX]

Supplementary Table 1. 104 differential gene pathways were screened between cluster1 with cluster3.

| ID | logFC | adj.P.Val |
| --- | --- | --- |
| KEGG_DRUG_METABOLISM_OTHER_ENZYMES  KEGG_BETA_ALANINE_METABOLISM  KEGG_GLYCEROLIPID_METABOLISM  KEGG_ONE_CARBON_POOL_BY_FOLATE  KEGG_TAURINE_AND_HYPOTAURINE_METABOLISM  KEGG_PROGESTERONE_MEDIATED_OOCYTE_MATURATION  KEGG_PENTOSE_AND_GLUCURONATE_INTERCONVERSIONS  KEGG_BLADDER_CANCER  KEGG_AMINOACYL_TRNA_BIOSYNTHESIS  KEGG_RETINOL_METABOLISM  KEGG_ARRHYTHMOGENIC_RIGHT_VENTRICULAR_CARDIOMYOPATHY_ARVC  KEGG_FRUCTOSE_AND_MANNOSE_METABOLISM  KEGG_PURINE_METABOLISM  KEGG_DORSO_VENTRAL_AXIS_FORMATION  KEGG_ALANINE_ASPARTATE_AND_GLUTAMATE_METABOLISM  KEGG_ADHERENS_JUNCTION  KEGG_ENDOMETRIAL_CANCER  KEGG_GNRH_SIGNALING_PATHWAY  KEGG_TRYPTOPHAN_METABOLISM  KEGG_TYROSINE_METABOLISM  KEGG_CELL_CYCLE  KEGG_HEDGEHOG_SIGNALING_PATHWAY  KEGG_BUTANOATE_METABOLISM  KEGG_VASCULAR_SMOOTH_MUSCLE_CONTRACTION  KEGG_GLYCINE_SERINE_AND_THREONINE_METABOLISM  KEGG_PORPHYRIN_AND_CHLOROPHYLL_METABOLISM  KEGG_GLYCEROPHOSPHOLIPID_METABOLISM  KEGG_FOLATE_BIOSYNTHESIS  KEGG_PENTOSE_PHOSPHATE_PATHWAY  KEGG_STARCH_AND_SUCROSE_METABOLISM  KEGG_PYRIMIDINE_METABOLISM  KEGG_ETHER_LIPID_METABOLISM  KEGG_FC_EPSILON_RI_SIGNALING_PATHWAY  KEGG_P53_SIGNALING_PATHWAY  KEGG_ENDOCYTOSIS  KEGG_ARGININE_AND_PROLINE_METABOLISM  KEGG_OLFACTORY_TRANSDUCTION  KEGG_SMALL_CELL_LUNG_CANCER  KEGG_HISTIDINE_METABOLISM  KEGG_SELENOAMINO_ACID_METABOLISM  KEGG_TIGHT_JUNCTION  KEGG_METABOLISM_OF_XENOBIOTICS_BY_CYTOCHROME_P450  KEGG_DRUG_METABOLISM_CYTOCHROME_P450  KEGG_PATHOGENIC_ESCHERICHIA_COLI_INFECTION  KEGG_CARDIAC_MUSCLE_CONTRACTION  KEGG_RIG_I_LIKE_RECEPTOR_SIGNALING_PATHWAY  KEGG_PANCREATIC_CANCER  KEGG_CHRONIC_MYELOID_LEUKEMIA  KEGG_MATURITY_ONSET_DIABETES_OF_THE_YOUNG  KEGG_N_GLYCAN_BIOSYNTHESIS  KEGG_T_CELL_RECEPTOR_SIGNALING_PATHWAY  KEGG_HYPERTROPHIC_CARDIOMYOPATHY_HCM  KEGG_DILATED_CARDIOMYOPATHY  KEGG_OOCYTE_MEIOSIS  KEGG_GLYCOLYSIS_GLUCONEOGENESIS  KEGG_TOLL_LIKE_RECEPTOR_SIGNALING_PATHWAY  KEGG_HEMATOPOIETIC_CELL_LINEAGE  KEGG_ANTIGEN_PROCESSING_AND_PRESENTATION  KEGG_VIRAL_MYOCARDITIS  KEGG_JAK_STAT_SIGNALING_PATHWAY  KEGG_OXIDATIVE_PHOSPHORYLATION  KEGG_NEUROACTIVE_LIGAND_RECEPTOR_INTERACTION  KEGG_COMPLEMENT_AND_COAGULATION_CASCADES  KEGG_PROSTATE_CANCER  KEGG_FOCAL_ADHESION  KEGG_HUNTINGTONS_DISEASE  KEGG_PRIMARY_IMMUNODEFICIENCY  KEGG_PEROXISOME  KEGG_WNT_SIGNALING_PATHWAY  KEGG_AXON_GUIDANCE  KEGG_CALCIUM_SIGNALING_PATHWAY  KEGG_AMINO_SUGAR_AND_NUCLEOTIDE_SUGAR_METABOLISM  KEGG_STEROID_BIOSYNTHESIS  KEGG_CYSTEINE_AND_METHIONINE_METABOLISM  KEGG_NEUROTROPHIN_SIGNALING_PATHWAY  KEGG_TGF_BETA_SIGNALING_PATHWAY  KEGG_NICOTINATE_AND_NICOTINAMIDE_METABOLISM  KEGG_ABC_TRANSPORTERS  KEGG_BIOSYNTHESIS_OF_UNSATURATED_FATTY_ACIDS  KEGG_CITRATE_CYCLE_TCA_CYCLE  KEGG_VASOPRESSIN_REGULATED_WATER_REABSORPTION  KEGG_TYPE_II_DIABETES_MELLITUS  KEGG_ALDOSTERONE_REGULATED_SODIUM_REABSORPTION  KEGG_O_GLYCAN_BIOSYNTHESIS  KEGG_MTOR_SIGNALING_PATHWAY  KEGG_VEGF_SIGNALING_PATHWAY  KEGG_VALINE_LEUCINE_AND_ISOLEUCINE_DEGRADATION  KEGG_NOTCH_SIGNALING_PATHWAY  KEGG_INSULIN_SIGNALING_PATHWAY  KEGG_PROPANOATE_METABOLISM  KEGG_LONG_TERM_POTENTIATION  KEGG_LONG_TERM_DEPRESSION  KEGG_REGULATION_OF_AUTOPHAGY  KEGG_ERBB_SIGNALING_PATHWAY  KEGG_INOSITOL_PHOSPHATE_METABOLISM  KEGG_VIBRIO_CHOLERAE_INFECTION  KEGG_GLYCOSYLPHOSPHATIDYLINOSITOL_GPI_ANCHOR_BIOSYNTHESIS  KEGG_SPHINGOLIPID_METABOLISM  KEGG_GLYCOSAMINOGLYCAN_DEGRADATION  KEGG_GLYCOSPHINGOLIPID_BIOSYNTHESIS_GLOBO_SERIES  KEGG_GLYCOSPHINGOLIPID_BIOSYNTHESIS_GANGLIO_SERIES  KEGG_RNA_DEGRADATION  KEGG_GLYCOSAMINOGLYCAN_BIOSYNTHESIS_CHONDROITIN_SULFATE | 0.780567  0.778211  0.773805  0.740851  0.726861  0.709506  0.705083  0.673998  0.664672  0.655506  0.645713  0.640273  0.629166  0.607361  0.588867  0.579210  0.579210  0.564840  0.527899  0.509264  0.506106  0.504317  0.503304  0.497911  0.467711  0.467711  0.453300  0.448455  0.442414  0.442414  0.437879  0.432916  0.408259  0.369801  0.369635  0.356099  0.341547  0.338123  0.327910  0.327910  0.318473  0.306319  0.306319  0.293131  0.284853  0.263063  0.263063  0.263063  0.245962  0.242681  0.233499  0.229143  0.229143  0.219498  0.207313  0.181703  0.164927  0.155896  0.142285  -0.125367  -0.152682  -0.157078  -0.168569  -0.183108  -0.219848  -0.233984  -0.241808  -0.280965  -0.281449  -0.297999  -0.324675  -0.339871  -0.340723  -0.406785  -0.410711  -0.413141  -0.414417  -0.418760  -0.422016  -0.425125  -0.494245  -0.512869  -0.514184  -0.543149  -0.543818  -0.552273  -0.570204  -0.595826  -0.612957  -0.618190  -0.639192  -0.639192  -0.646950  -0.666885  -0.671173  -0.703739  -0.704778  -0.725635  -0.727527  -0.727527  -0.727527  -0.733968  -0.813787 | \| 1.27E-14 \| \| --- \| \| 7.85E-11 \| \| 1.61E-12 \| \| 2.59E-09 \| \| 2.88E-09 \| \| 1.61E-12 \| \| 1.86E-09 \| \| 3.43E-08 \| \| 1.19E-07 \| \| 1.72E-08 \| \| 8.17E-09 \| \| 1.50E-07 \| \| 3.60E-14 \| \| 1.99E-07 \| \| 3.74E-07 \| \| 3.16E-06 \| \| 3.16E-06 \| \| 3.48E-08 \| \| 2.83E-05 \| \| 1.19E-07 \| \| 1.32E-10 \| \| 6.43E-05 \| \| 6.68E-05 \| \| 8.80E-05 \| \| 0.000239 \| \| 0.000239 \| \| 6.85E-05 \| \| 0.000505 \| \| 0.000342 \| \| 0.000342 \| \| 5.85E-05 \| \| 0.000574 \| \| 0.001517 \| \| 4.46E-06 \| \| 4.20E-08 \| \| 0.00028 \| \| 0.000362 \| \| 1.89E-05 \| \| 0.005892 \| \| 0.005892 \| \| 3.43E-08 \| \| 0.002292 \| \| 0.002292 \| \| 0.00204 \| \| 0.019084 \| \| 0.026689 \| \| 0.026689 \| \| 0.026689 \| \| 0.043172 \| \| 0.011425 \| \| 0.000323 \| \| 0.046543 \| \| 0.046543 \| \| 0.020248 \| \| 0.022407 \| \| 0.041911 \| \| 0.010606 \| \| 0.032283 \| \| 0.048896 \| \| 0.048642 \| \| 0.03919 \| \| 0.012762 \| \| 0.013662 \| \| 0.02746 \| \| 0.00276 \| \| 0.002302 \| \| 0.002418 \| \| 0.00132 \| \| 0.030919 \| \| 0.000492 \| \| 3.98E-05 \| \| 0.000663 \| \| 0.009683 \| \| 0.001564 \| \| 1.34E-05 \| \| 1.71E-06 \| \| 0.000163 \| \| 0.001034 \| \| 0.000876 \| \| 0.000838 \| \| 2.82E-05 \| \| 1.66E-05 \| \| 4.83E-05 \| \| 9.17E-07 \| \| 2.90E-07 \| \| 1.28E-05 \| \| 9.17E-09 \| \| 2.13E-06 \| \| 3.21E-12 \| \| 2.73E-08 \| \| 2.42E-07 \| \| 2.42E-07 \| \| 1.47E-07 \| \| 1.58E-08 \| \| 5.66E-08 \| \| 1.86E-09 \| \| 1.58E-08 \| \| 4.75E-09 \| \| 4.13E-09 \| \| 4.13E-09 \| \| 4.13E-09 \| \| 2.70E-09 \| \| 4.71E-11 \| |
