# Supplementary material for: Comprehensive Analysis of Molecular Subtypes and Hub Genes of Sepsis by Gene Expression Profiles
Source: Front Genet. 2022 Aug 12;13:884762. doi: 10.3389/fgene.2022.884762 (PMC9412106; doi:10.3389/fgene.2022.884762)
Supplement: Supplementary file 2 [file Table2.DOCX]

Supplementary Table 2. 90 differential gene pathways were screened between cluster1 with cluster4.

| ID | logFC | adj.P.Val |
| --- | --- | --- |
| KEGG_OXIDATIVE_PHOSPHORYLATION  KEGG_VIBRIO_CHOLERAE_INFECTION  KEGG_PEROXISOME  KEGG_ARGININE_AND_PROLINE_METABOLISM  KEGG_FATTY_ACID_METABOLISM  KEGG_DNA_REPLICATION  KEGG_TIGHT_JUNCTION  KEGG_LYSOSOME  KEGG_O_GLYCAN_BIOSYNTHESIS  KEGG_AUTOIMMUNE_THYROID_DISEASE  KEGG_ALLOGRAFT_REJECTION  KEGG_VIRAL_MYOCARDITIS  KEGG_INOSITOL_PHOSPHATE_METABOLISM  KEGG_PHOSPHATIDYLINOSITOL_SIGNALING_SYSTEM  KEGG_ABC_TRANSPORTERS  KEGG_PURINE_METABOLISM  KEGG_CELL_ADHESION_MOLECULES_CAMS  KEGG_OOCYTE_MEIOSIS  KEGG_PROGESTERONE_MEDIATED_OOCYTE_MATURATION  KEGG_PROXIMAL_TUBULE_BICARBONATE_RECLAMATION  KEGG_NITROGEN_METABOLISM  KEGG_GLYCEROPHOSPHOLIPID_METABOLISM  KEGG_SYSTEMIC_LUPUS_ERYTHEMATOSUS  KEGG_ARRHYTHMOGENIC_RIGHT_VENTRICULAR_CARDIOMYOPATHY_ARVC  KEGG_ADHERENS_JUNCTION  KEGG_ENDOMETRIAL_CANCER  KEGG_EPITHELIAL_CELL_SIGNALING_IN_HELICOBACTER_PYLORI_INFECTION  KEGG_ENDOCYTOSIS  KEGG_CELL_CYCLE  KEGG_TYPE_I_DIABETES_MELLITUS  KEGG_GRAFT_VERSUS_HOST_DISEASE  KEGG_ADIPOCYTOKINE_SIGNALING_PATHWAY  KEGG_GLUTATHIONE_METABOLISM  KEGG_JAK_STAT_SIGNALING_PATHWAY  KEGG_PPAR_SIGNALING_PATHWAY  KEGG_LEUKOCYTE_TRANSENDOTHELIAL_MIGRATION  KEGG_ARACHIDONIC_ACID_METABOLISM  KEGG_UBIQUITIN_MEDIATED_PROTEOLYSIS  KEGG_ASTHMA  KEGG_T_CELL_RECEPTOR_SIGNALING_PATHWAY  KEGG_LEISHMANIA_INFECTION  KEGG_PRION_DISEASES  KEGG_CYTOKINE_CYTOKINE_RECEPTOR_INTERACTION  KEGG_CALCIUM_SIGNALING_PATHWAY  KEGG_FC_GAMMA_R_MEDIATED_PHAGOCYTOSIS  KEGG_NEUROACTIVE_LIGAND_RECEPTOR_INTERACTION  KEGG_CITRATE_CYCLE_TCA_CYCLE  KEGG_GLYCINE_SERINE_AND_THREONINE_METABOLISM  KEGG_CYTOSOLIC_DNA_SENSING_PATHWAY  KEGG_COMPLEMENT_AND_COAGULATION_CASCADES  KEGG_CHEMOKINE_SIGNALING_PATHWAY  KEGG_RENAL_CELL_CARCINOMA  KEGG_NUCLEOTIDE_EXCISION_REPAIR  KEGG_PROPANOATE_METABOLISM  KEGG_NICOTINATE_AND_NICOTINAMIDE_METABOLISM  KEGG_DRUG_METABOLISM_CYTOCHROME_P450  KEGG_GLYCOSAMINOGLYCAN_BIOSYNTHESIS_HEPARAN_SULFATE  KEGG_VALINE_LEUCINE_AND_ISOLEUCINE_DEGRADATION  KEGG_TERPENOID_BACKBONE_BIOSYNTHESIS  KEGG_REGULATION_OF_ACTIN_CYTOSKELETON  KEGG_GLYCOSAMINOGLYCAN_BIOSYNTHESIS_CHONDROITIN_SULFATE  KEGG_NOTCH_SIGNALING_PATHWAY  KEGG_TGF_BETA_SIGNALING_PATHWAY  KEGG_FC_EPSILON_RI_SIGNALING_PATHWAY  KEGG_NOD_LIKE_RECEPTOR_SIGNALING_PATHWAY  KEGG_ACUTE_MYELOID_LEUKEMIA  KEGG_ERBB_SIGNALING_PATHWAY  KEGG_MTOR_SIGNALING_PATHWAY  KEGG_RIG_I_LIKE_RECEPTOR_SIGNALING_PATHWAY  KEGG_APOPTOSIS  KEGG_PROSTATE_CANCER  KEGG_ECM_RECEPTOR_INTERACTION  KEGG_PYRUVATE_METABOLISM  KEGG_STEROID_BIOSYNTHESIS  KEGG_GLYCOLYSIS_GLUCONEOGENESIS  KEGG_VASOPRESSIN_REGULATED_WATER_REABSORPTION  KEGG_FOCAL_ADHESION  KEGG_GALACTOSE_METABOLISM  KEGG_RNA_DEGRADATION  KEGG_MAPK_SIGNALING_PATHWAY  KEGG_PENTOSE_PHOSPHATE_PATHWAY  KEGG_STARCH_AND_SUCROSE_METABOLISM  KEGG_ALZHEIMERS_DISEASE  KEGG_HUNTINGTONS_DISEASE  KEGG_GLYCOSAMINOGLYCAN_DEGRADATION  KEGG_N_GLYCAN_BIOSYNTHESIS  KEGG_INSULIN_SIGNALING_PATHWAY  KEGG_AMINO_SUGAR_AND_NUCLEOTIDE_SUGAR_METABOLISM  KEGG_REGULATION_OF_AUTOPHAGY  KEGG_NEUROTROPHIN_SIGNALING_PATHWAY | 0.717308  0.717308  0.683635  0.659438  0.627129  0.615844  0.606591  0.596454  0.592480  0.584328  0.584328  0.584328  0.559296  0.559296  0.546576  0.518409  0.474204  0.468293  0.468293  0.454469  0.454469  0.447440  0.440384  0.438246  0.438246  0.438246  0.388668  0.372432  0.354897  0.352273  0.352273  0.339914  0.327450  0.319498  0.296303  0.285243  0.271691  0.255361  0.243699  0.193177  0.185942  0.180923  -0.119925  -0.190875  -0.249088  -0.249860  -0.271444  -0.271444  -0.273060  -0.274482  -0.280105  -0.303526  -0.309937  -0.346480  -0.346511  -0.348551  -0.358467  -0.358646  -0.362814  -0.400020  -0.402082  -0.404106  -0.410231  -0.418035  -0.425778  -0.440988  -0.449776  -0.449776  -0.456171  -0.456948  -0.457615  -0.462313  -0.465757  -0.470982  -0.491987  -0.527597  -0.540241  -0.546293  -0.549372  -0.552787  -0.561849  -0.561849  -0.565466  -0.577884  -0.596211  -0.599118  -0.615713  -0.661325  -0.675728  -0.677345 | 3.79E-13  3.79E-13  1.87E-12  1.87E-12  5.77E-10  3.98E-09  2.34E-10  1.20E-09  1.06E-08  2.45E-09  2.45E-09  2.45E-09  9.10E-08  9.10E-08  1.70E-07  1.16E-09  9.11E-12  8.28E-06  8.28E-06  2.13E-06  2.13E-06  2.00E-05  3.98E-09  1.76E-05  1.76E-05  1.76E-05  2.13E-06  6.39E-06  5.07E-05  2.00E-05  2.00E-05  0.000156  0.000056  0.000175  0.000417  0.000048  0.000221  0.000599  0.005082  0.000864  0.043246  0.012470  0.035703  0.021352  0.018839  0.000018  0.008887  0.008887  0.006208  0.001007  0.000003  0.004452  0.003702  0.000905  0.001005  0.000894  0.000599  0.000123  0.000534  0.000162  0.000146  0.000160  9.25E-05  7.71E-05  1.50E-07  2.36E-06  1.76E-05  1.76E-05  2.53E-06  1.70E-07  1.33E-07  7.75E-07  8.98E-07  7.56E-06  7.75E-07  5.78E-07  3.83E-10  1.80E-07  1.50E-07  4.04E-13  5.27E-08  5.27E-08  4.04E-13  2.67E-08  1.14E-08  8.76E-11  6.03E-11  5.21E-12  4.91E-13  6.40E-13 |
