# Supplementary material for: Comprehensive Analysis of Molecular Subtypes and Hub Genes of Sepsis by Gene Expression Profiles
Source: Front Genet. 2022 Aug 12;13:884762. doi: 10.3389/fgene.2022.884762 (PMC9412106; doi:10.3389/fgene.2022.884762)
Supplement: Supplementary file 3 [file Table3.DOCX]

Supplementary Table 3. 110 differential gene pathways were screened between cluster3 with cluster4.

| ID | logFC | adj.P.Val |
| --- | --- | --- |
| KEGG_FATTY_ACID_METABOLISM  KEGG_NOTCH_SIGNALING_PATHWAY  KEGG_VEGF_SIGNALING_PATHWAY  KEGG_PROPANOATE_METABOLISM  KEGG_O_GLYCAN_BIOSYNTHESIS  KEGG_LYSINE_DEGRADATION  KEGG_RNA_DEGRADATION  KEGG_DNA_REPLICATION  KEGG_TYPE_II_DIABETES_MELLITUS  KEGG_LONG_TERM_DEPRESSION  KEGG_SPHINGOLIPID_METABOLISM  KEGG_CYSTEINE_AND_METHIONINE_METABOLISM  KEGG_LONG_TERM_POTENTIATION  KEGG_BUTANOATE_METABOLISM  KEGG_TERPENOID_BACKBONE_BIOSYNTHESIS  KEGG_ABC_TRANSPORTERS  KEGG_GLYCOSYLPHOSPHATIDYLINOSITOL_GPI_ANCHOR_BIOSYNTHESIS  KEGG_STEROID_BIOSYNTHESIS  KEGG_BASAL_CELL_CARCINOMA  KEGG_GLYCOSPHINGOLIPID_BIOSYNTHESIS_GLOBO_SERIES  KEGG_GLYCOSPHINGOLIPID_BIOSYNTHESIS_GANGLIO_SERIES  KEGG_BASAL_TRANSCRIPTION_FACTORS  KEGG_GRAFT_VERSUS_HOST_DISEASE  KEGG_INSULIN_SIGNALING_PATHWAY  KEGG_AXON_GUIDANCE  KEGG_BIOSYNTHESIS_OF_UNSATURATED_FATTY_ACIDS  KEGG_VALINE_LEUCINE_AND_ISOLEUCINE_DEGRADATION  KEGG_INOSITOL_PHOSPHATE_METABOLISM  KEGG_APOPTOSIS  KEGG_ALLOGRAFT_REJECTION  KEGG_NICOTINATE_AND_NICOTINAMIDE_METABOLISM  KEGG_AMYOTROPHIC_LATERAL_SCLEROSIS_ALS  KEGG_TYPE_I_DIABETES_MELLITUS  KEGG_ARACHIDONIC_ACID_METABOLISM  KEGG_CELL_ADHESION_MOLECULES_CAMS  KEGG_PHENYLALANINE_METABOLISM  KEGG_UBIQUITIN_MEDIATED_PROTEOLYSIS  KEGG_AMINO_SUGAR_AND_NUCLEOTIDE_SUGAR_METABOLISM  KEGG_ANTIGEN_PROCESSING_AND_PRESENTATION  KEGG_TGF_BETA_SIGNALING_PATHWAY  KEGG_ARRHYTHMOGENIC_RIGHT_VENTRICULAR_CARDIOMYOPATHY_ARVC  KEGG_SMALL_CELL_LUNG_CANCER  KEGG_ECM_RECEPTOR_INTERACTION  KEGG_NEUROACTIVE_LIGAND_RECEPTOR_INTERACTION  KEGG_HYPERTROPHIC_CARDIOMYOPATHY_HCM  KEGG_GLUTATHIONE_METABOLISM  KEGG_WNT_SIGNALING_PATHWAY  KEGG_SPLICEOSOME  KEGG_GLYCOSAMINOGLYCAN_BIOSYNTHESIS_CHONDROITIN_SULFATE  KEGG_AUTOIMMUNE_THYROID_DISEASE  KEGG_VIRAL_MYOCARDITIS  KEGG_SYSTEMIC_LUPUS_ERYTHEMATOSUS  KEGG_VIBRIO_CHOLERAE_INFECTION  KEGG_PEROXISOME  KEGG_PRION_DISEASES  KEGG_JAK_STAT_SIGNALING_PATHWAY  KEGG_ADHERENS_JUNCTION  KEGG_CYTOKINE_CYTOKINE_RECEPTOR_INTERACTION  KEGG_PATHOGENIC_ESCHERICHIA_COLI_INFECTION  KEGG_OLFACTORY_TRANSDUCTION  KEGG_METABOLISM_OF_XENOBIOTICS_BY_CYTOCHROME_P450  KEGG_MAPK_SIGNALING_PATHWAY  KEGG_ERBB_SIGNALING_PATHWAY  KEGG_PURINE_METABOLISM  KEGG_CIRCADIAN_RHYTHM_MAMMAL  KEGG_CHEMOKINE_SIGNALING_PATHWAY  KEGG_T_CELL_RECEPTOR_SIGNALING_PATHWAY  KEGG_CELL_CYCLE  KEGG_EPITHELIAL_CELL_SIGNALING_IN_HELICOBACTER_PYLORI_INFECTION  KEGG_LYSOSOME  KEGG_SELENOAMINO_ACID_METABOLISM  KEGG_ENDOMETRIAL_CANCER  KEGG_PROSTATE_CANCER  KEGG_TOLL_LIKE_RECEPTOR_SIGNALING_PATHWAY  KEGG_GAP_JUNCTION  KEGG_DRUG_METABOLISM_CYTOCHROME_P450  KEGG_NUCLEOTIDE_EXCISION_REPAIR  KEGG_GLYCEROLIPID_METABOLISM  KEGG_ACUTE_MYELOID_LEUKEMIA  KEGG_THYROID_CANCER  KEGG_ETHER_LIPID_METABOLISM  KEGG_N_GLYCAN_BIOSYNTHESIS  KEGG_GLYCOSPHINGOLIPID_BIOSYNTHESIS_LACTO_AND_NEOLACTO_SERIES  KEGG_PANCREATIC_CANCER  KEGG_MATURITY_ONSET_DIABETES_OF_THE_YOUNG  KEGG_COLORECTAL_CANCER  KEGG_PORPHYRIN_AND_CHLOROPHYLL_METABOLISM  KEGG_NON_SMALL_CELL_LUNG_CANCER  KEGG_CYTOSOLIC_DNA_SENSING_PATHWAY  KEGG_PROGESTERONE_MEDIATED_OOCYTE_MATURATION  KEGG_NON_HOMOLOGOUS_END_JOINING  KEGG_RIBOFLAVIN_METABOLISM  KEGG_TRYPTOPHAN_METABOLISM  KEGG_GLYCINE_SERINE_AND_THREONINE_METABOLISM  KEGG_VASCULAR_SMOOTH_MUSCLE_CONTRACTION  KEGG_FC_EPSILON_RI_SIGNALING_PATHWAY  KEGG_GNRH_SIGNALING_PATHWAY  KEGG_BETA_ALANINE_METABOLISM  KEGG_FOLATE_BIOSYNTHESIS  KEGG_AMINOACYL_TRNA_BIOSYNTHESIS  KEGG_LINOLEIC_ACID_METABOLISM  KEGG_VASOPRESSIN_REGULATED_WATER_REABSORPTION  KEGG_PENTOSE_PHOSPHATE_PATHWAY  KEGG_ONE_CARBON_POOL_BY_FOLATE  KEGG_PYRUVATE_METABOLISM  KEGG_RETINOL_METABOLISM  KEGG_DRUG_METABOLISM_OTHER_ENZYMES  KEGG_HISTIDINE_METABOLISM  KEGG_REGULATION_OF_AUTOPHAGY  KEGG_PENTOSE_AND_GLUCURONATE_INTERCONVERSIONS | 0.959824  0.872807  0.866592  0.854446  0.834559  0.832294  0.800155  0.797771  0.796051  0.793998  0.775749  0.774043  0.756623  0.731781  0.731781  0.705719  0.680144  0.649087  0.625595  0.625009  0.625009  0.606435  0.595715  0.555504  0.509886  0.505593  0.503499  0.466721  0.464940  0.463108  0.456673  0.440397  0.386880  0.362726  0.360212  0.351962  0.330400  0.275772  0.266578  0.250816  0.237998  0.235026  0.233220  0.230147  0.228202  0.227279  0.226465  0.225357  0.213369  0.207571  0.207443  0.185472  0.181800  0.170051  0.162343  0.157143  -0.185781  -0.190526  -0.197809  -0.213485  -0.214305  -0.215106  -0.224556  -0.238659  -0.239214  -0.264892  -0.277672  -0.277785  -0.313419  -0.324059  -0.324967  -0.339120  -0.352320  -0.386409  -0.411588  -0.467135  -0.476880  -0.481886  -0.538426  -0.546168  -0.618829  -0.637578  -0.653509  -0.653772  -0.657433  -0.660718  -0.661499  -0.692947  -0.695833  -0.704903  -0.732705  -0.761944  -0.785016  -0.785732  -0.817741  -0.824302  -0.833539  -0.844076  -0.847858  -0.852962  -0.853221  -0.853736  -0.884935  -0.911933  -0.917764  -0.925668  -0.943485  -0.962110  -0.962430  -1.004071 | \| 4.34E-18 \| \| --- \| \| 6.81E-12 \| \| 5.25E-12 \| \| 8.87E-12 \| \| 1.59E-11 \| \| 1.25E-11 \| \| 6.71E-14 \| \| 2.15E-11 \| \| 9.59E-12 \| \| 1.34E-09 \| \| 1.64E-10 \| \| 1.48E-09 \| \| 4.81E-09 \| \| 2.83E-08 \| \| 2.83E-08 \| \| 1.34E-09 \| \| 8.42E-07 \| \| 1.72E-06 \| \| 7.51E-06 \| \| 4.32E-06 \| \| 4.32E-06 \| \| 1.72E-05 \| \| 3.66E-06 \| \| 2.30E-11 \| \| 7.28E-13 \| \| 0.00059 \| \| 9.65E-08 \| \| 4.32E-06 \| \| 1.86E-06 \| \| 2.88E-05 \| \| 0.001689 \| \| 3.24E-05 \| \| 0.000333 \| \| 1.35E-05 \| \| 3.76E-06 \| \| 0.002697 \| \| 9.11E-06 \| \| 0.004485 \| \| 0.002396 \| \| 0.003611 \| \| 0.000116 \| \| 0.004251 \| \| 0.010212 \| \| 8.80E-05 \| \| 0.002582 \| \| 0.001807 \| \| 0.003848 \| \| 0.011346 \| \| 0.022162 \| \| 0.024669 \| \| 0.010283 \| \| 0.011252 \| \| 0.016519 \| \| 0.030311 \| \| 0.043285 \| \| 0.007872 \| \| 0.035605 \| \| 0.000116 \| \| 0.022192 \| \| 0.003718 \| \| 0.018758 \| \| 3.37E-05 \| \| 0.002328 \| \| 0.008487 \| \| 0.029663 \| \| 9.95E-06 \| \| 6.50E-07 \| \| 1.40E-05 \| \| 7.09E-06 \| \| 4.09E-07 \| \| 0.011286 \| \| 0.008737 \| \| 0.001689 \| \| 1.32E-05 \| \| 0.000527 \| \| 7.70E-07 \| \| 7.45E-05 \| \| 1.70E-09 \| \| 0.000216 \| \| 0.000147 \| \| 1.05E-05 \| \| 5.49E-13 \| \| 2.85E-06 \| \| 4.09E-07 \| \| 3.89E-06 \| \| 3.03E-06 \| \| 3.76E-06 \| \| 6.87E-07 \| \| 1.52E-07 \| \| 9.31E-19 \| \| 6.42E-08 \| \| 1.68E-09 \| \| 2.80E-12 \| \| 1.13E-10 \| \| 2.72E-11 \| \| 2.65E-16 \| \| 2.91E-18 \| \| 4.76E-11 \| \| 7.05E-12 \| \| 1.59E-11 \| \| 6.81E-12 \| \| 1.77E-12 \| \| 5.25E-12 \| \| 1.90E-13 \| \| 4.39E-14 \| \| 6.25E-14 \| \| 9.67E-15 \| \| 4.87E-18 \| \| 1.81E-17 \| \| 2.17E-18 \| |
