# Supplementary material for: Comprehensive Analysis of Molecular Subtypes and Hub Genes of Sepsis by Gene Expression Profiles
Source: Front Genet. 2022 Aug 12;13:884762. doi: 10.3389/fgene.2022.884762 (PMC9412106; doi:10.3389/fgene.2022.884762)
Supplement: Supplementary file 4 [file Table4.DOCX]

f


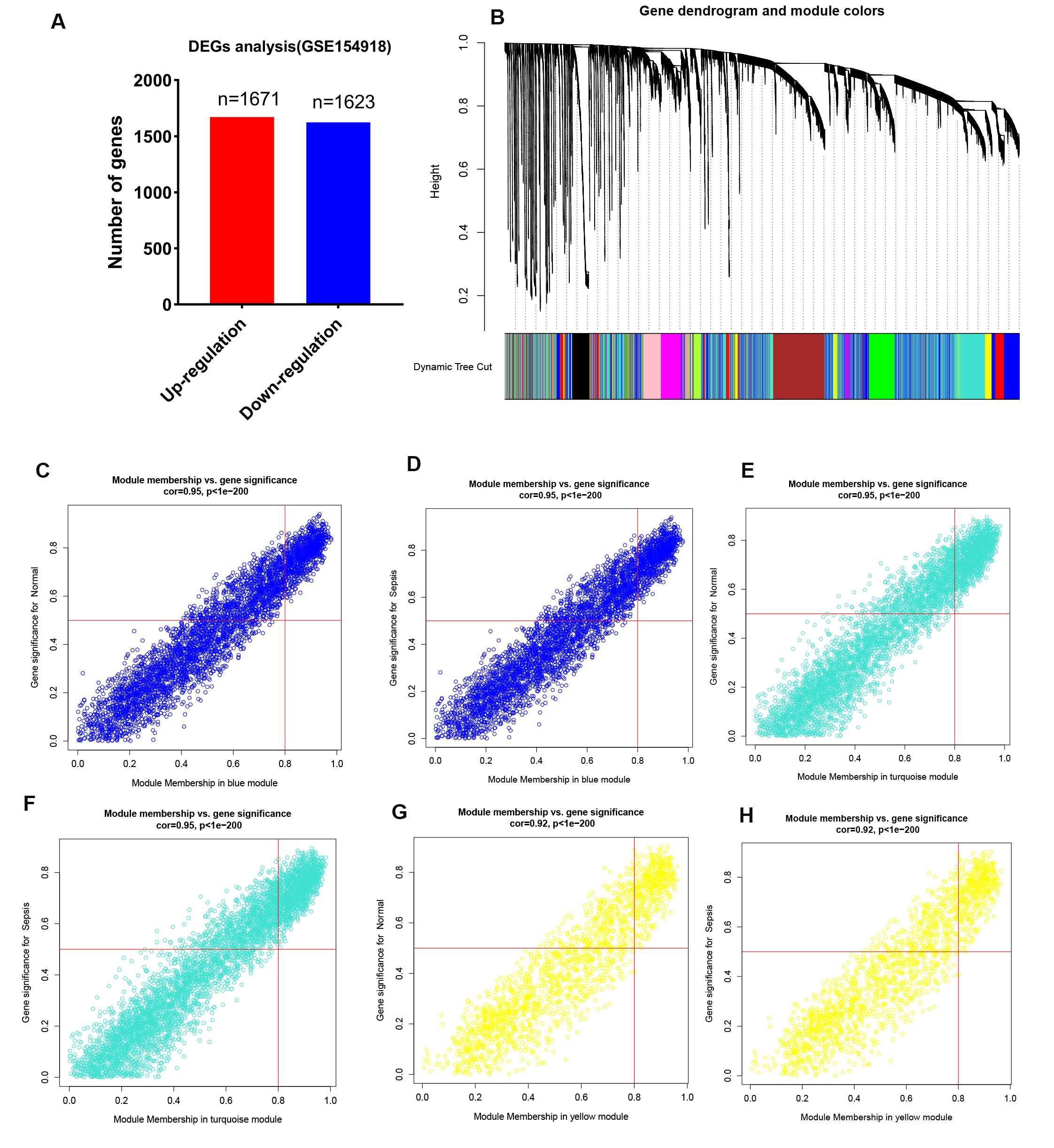


**FIGURE S1 GSE154918 dataset were analyzed by DEGs and WGCNA methods, respectively.** (A) Representative histogram of 1671 up-regulated DEGs and 1623 down-regulated DEGs. (B) clustering dendrogram: each color represents a specific co-expression module. The colored row below the tree diagram represents module colors. (C-H) The correlation analysis between blue, turquoise, and yellow modules with module-related genes. Normal and Sepsis group present the same scatter pattern.


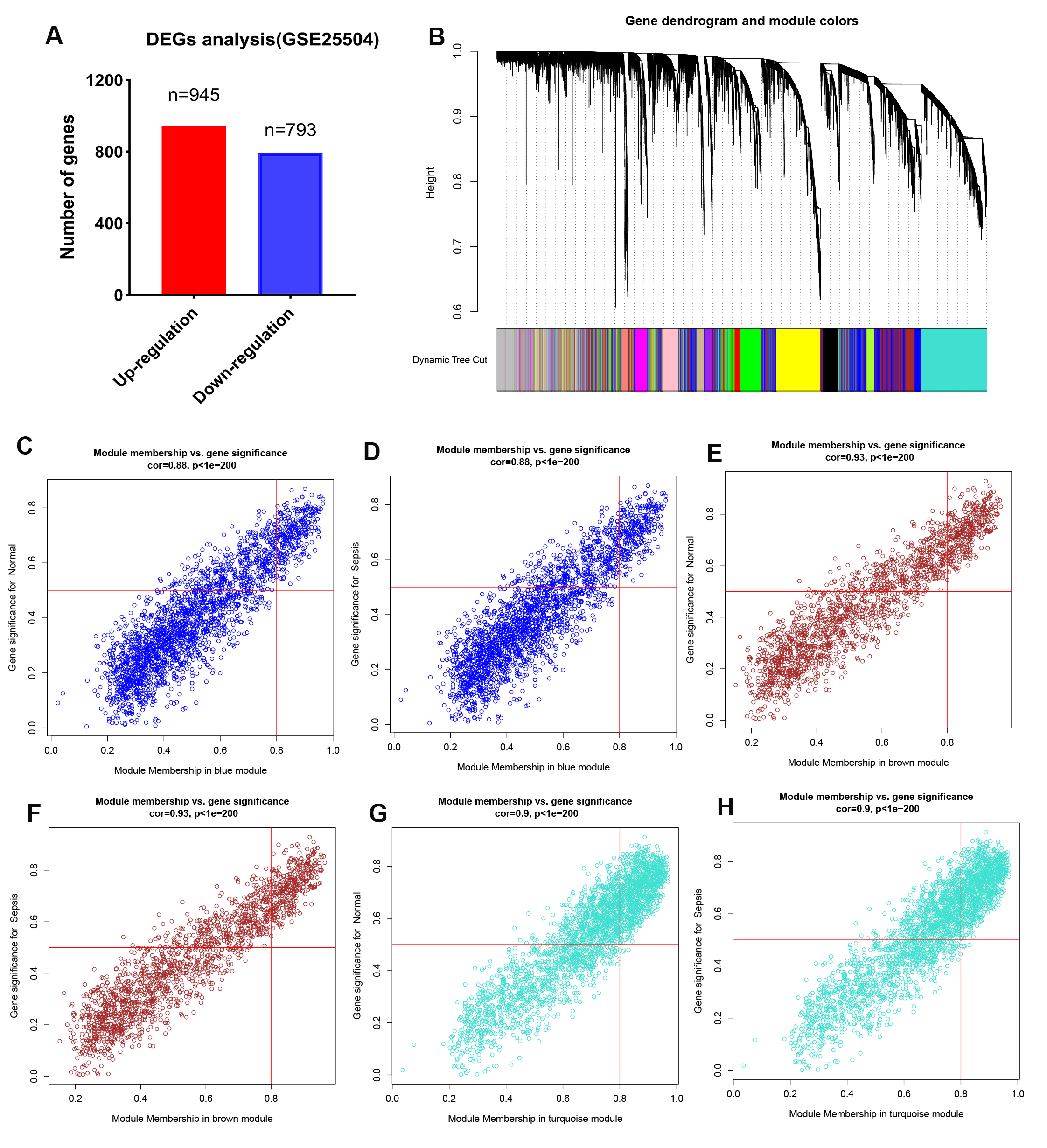


**FIGURE S2** **GSE25504 dataset were analyzed by DEGs and WGCNA methods, respectively.** (A) Representative histogram of 945 up-regulated DEGs and 793 down-regulated DEGs. (B) clustering dendrogram: each color represents a specific co-expression module. The colored row below the tree diagram represents module colors. (C-H) The correlation analysis between blue, brown, and turquoise modules with module-related genes. Normal and Sepsis group present the same scatter pattern.


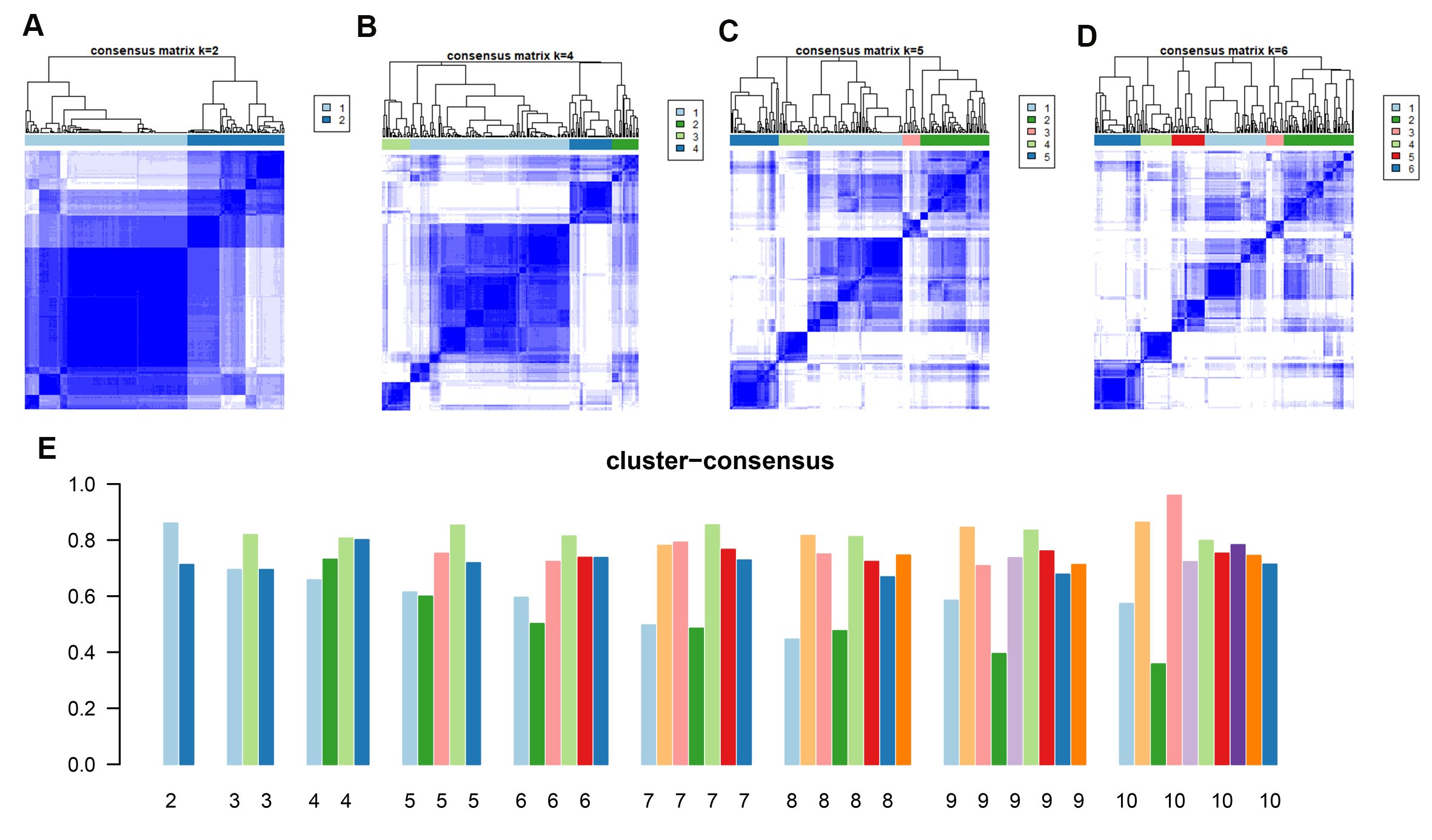


**FIGURE S3 Consensus clustering analysis for sepsis.** (A-D) Consensus clustering matrix for k=2, k=4, k=5, k=6. (E) The score of consensus clustering.


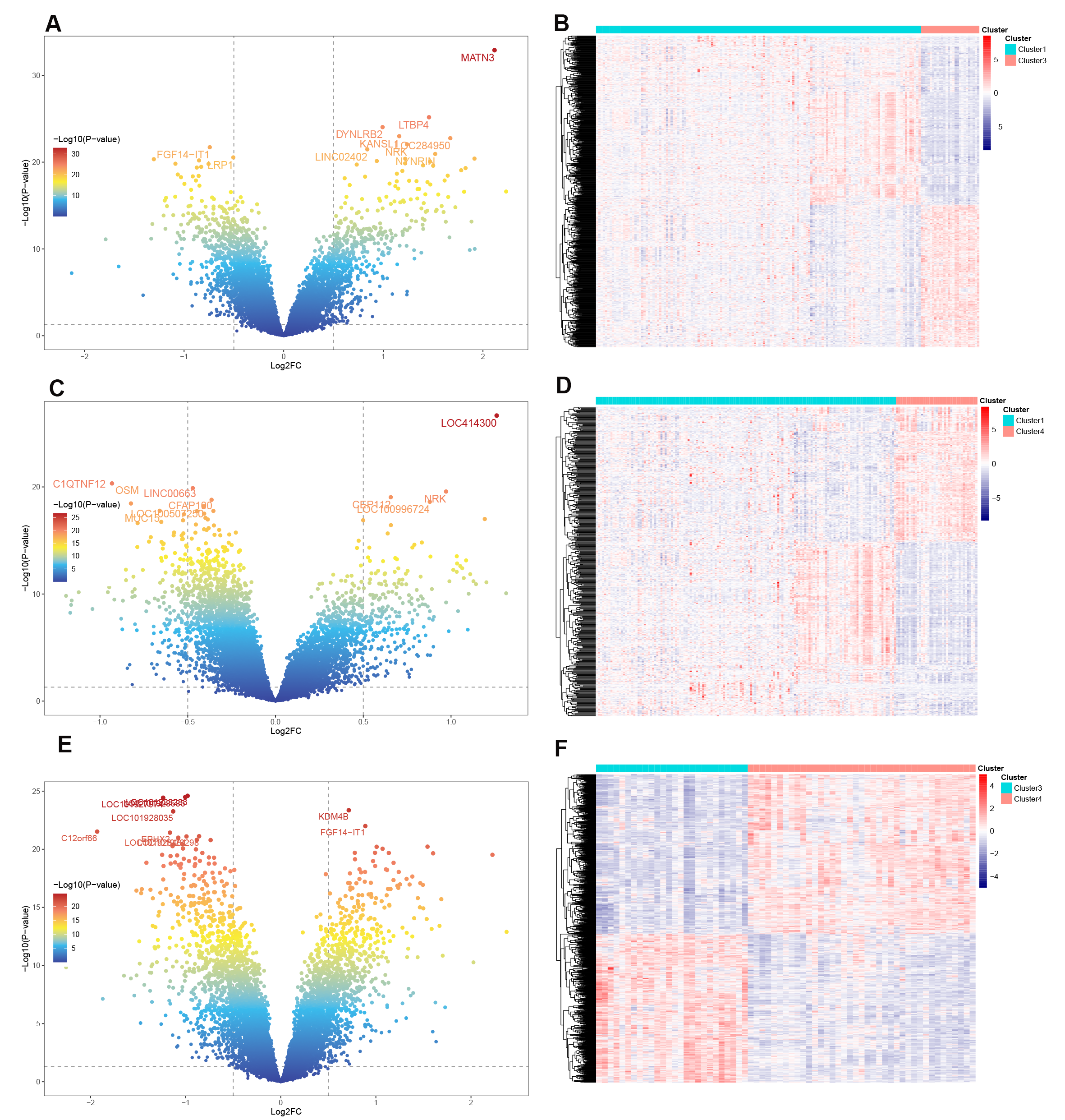


**FIGURE S4 Identification of DEGs among different sepsis molecular subtypes.** (A, B) Representative volcano plot and heat map of DEGs between Cluster1 and Cluster3. (C, D) Representative volcano plot and heat map of DEGs between Cluster1 and Cluster4. (E, F) Representative volcano plot and heat map of DEGs between Cluster3 and Cluster4.


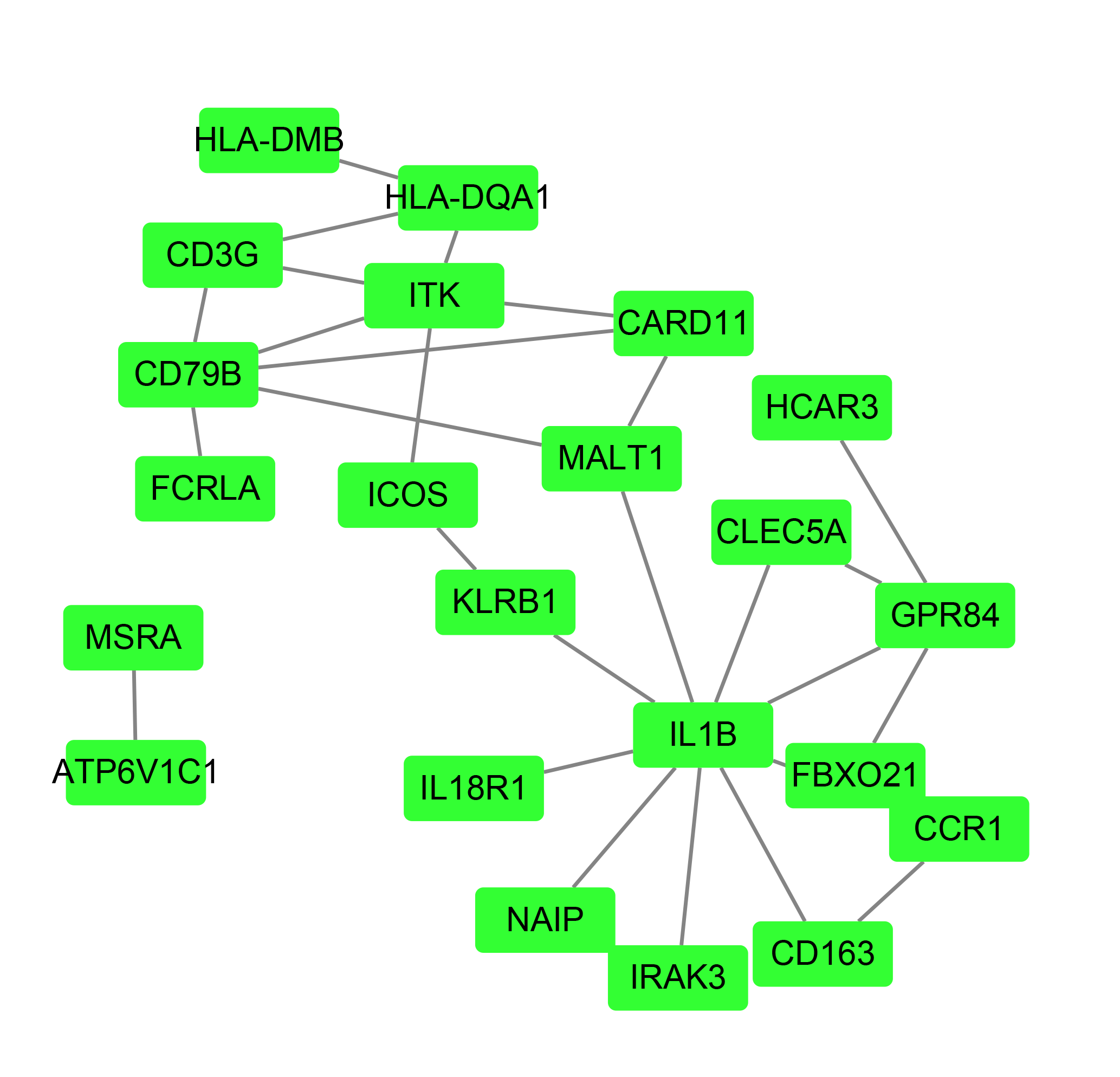


**FIGURE S5 Construction of PPI network.** Construction of hub network based on co-DEGs.
